# Soils and topography drive large and predictable shifts in canopy dynamics across tropical forest landscapes

**DOI:** 10.1111/nph.70300

**Published:** 2025-06-13

**Authors:** Beibei Zhang, Toby D. Jackson, David A. Coomes, David F. R. P. Burslem, Reuben Nilus, Paulo R. L. Bittencourt, David C. Bartholomew, Lucy Rowland, Fabian J. Fischer, Tommaso Jucker

**Affiliations:** ^1^ School of Biological Sciences University of Bristol 24 Tyndall Ave Bristol BS8 1TQ UK; ^2^ Department of Plant Sciences and Conservation Research Institute University of Cambridge Cambridge UK; ^3^ School of Biological Sciences University of Aberdeen Cruickshank Building Aberdeen AB24 3UU UK; ^4^ Sabah Forestry Department Forest Research Centre PO Box 1407 Sandakan 90715 Sabah Malaysia; ^5^ School of Earth and Environmental Sciences Cardiff University Main Building Cardiff CF10 3AT UK; ^6^ Department of Geography, Faculty of Environment Society and Economy University of Exeter Exeter UK; ^7^ Botanic Gardens Conservation International Descanso House, 199 Kew Road Richmond TW9 3BW UK

**Keywords:** canopy gaps, forest disturbance, forest dynamics, forest structure, LiDAR, remote sensing, soil nutrients, topography

## Abstract

- Tropical forests can vary enormously in their 3D structure and dynamics across surprisingly small spatial scales. However, the drivers that underpin this local‐scale variation in forest structure and dynamics remain poorly understood.
- We acquired repeat airborne laser scanning data across an old‐growth tropical forest landscape in Malaysian Borneo, characterized by a steep gradient in soil fertility and topography that gives rise to large variability in canopy 3D structure. Using this unique dataset, we explored how local‐scale variation in topography and forest structure shapes rates of gap formation, closure, and canopy growth across the landscape.
- We found that both canopy gains and losses were 2.5–4.7 times greater in low‐lying alluvial forests on fertile soils than in nearby nutrient‐depleted kerangas forests on hilltops. Moreover, we found that variation in canopy 3D structure and dynamics was tightly coupled across the landscape, with taller and more structurally heterogeneous canopies also experiencing faster rates of gap dynamics.
- Our study highlights the key role that soils and topography play in shaping the structural complexity and dynamics of tropical forest landscapes.

## Introduction

Whether or not intact tropical forests will continue to act as net carbon sinks into the future is increasingly uncertain, with evidence that increasing disturbance rates in some regions are compromising their long‐term capacity to sequester and store carbon (Qie *et al*., 2017; Hubau *et al*., 2020; Bauman *et al*., 2022; Csillik *et al*., 2024; Pan *et al*., 2024). Part of this uncertainty stems from the fact that tropical forest canopies vary enormously from place to place in their composition, structure, and dynamics (Sullivan *et al*., 2017; Lutz *et al*., 2018; Dalagnol *et al*., 2021; Piponiot *et al*., 2022; Li *et al*., 2023), all of which influence their susceptibility to climate change (Chen *et al*., 2024). At pan‐tropical scales, variation in canopy dynamics linked to disturbance and recovery processes reflects differences in climate, biogeography, and disturbance regimes among tropical regions (Gorgens *et al*., 2021; Muller‐Landau *et al*., 2021; Li *et al*., 2023; Ankori‐Karlinsky *et al*., 2024; Jackson *et al*., 2024b). But tropical forest canopies can also vary dramatically at local scales, with differences within landscapes often comparable to those observed across entire biogeographic regions (Werner & Homeier, 2015; Jucker *et al*., 2018; Oliveira *et al*., 2019; Muscarella *et al*., 2020; Dalagnol *et al*., 2021; Bittencourt *et al*., 2022; Cushman *et al*., 2022). Understanding what causes the structure and dynamics of tropical forest canopies to vary so much at local scales is critical if we are to track how these ecosystems are responding to rapid global change (McDowell *et al*., 2020).

There are numerous reasons why forest canopy structure and dynamics vary within tropical landscapes, but soils, topography, and water availability likely play particularly important roles (Quesada *et al*., 2012; Werner & Homeier, 2015; Jucker *et al*., 2018; Muscarella *et al*., 2020; Cushman *et al*., 2022; Sousa *et al*., 2022). Soil fertility—especially the availability of phosphorus, nitrogen, and macro‐nutrient cations—is a major ecological filter in tropical forests, shaping community composition, species traits, and their demographic rates (Quesada *et al*., 2012; Werner & Homeier, 2015; Chadwick & Asner, 2018; Bongalov *et al*., 2019; Muller‐Landau *et al*., 2021; Bartholomew *et al*., 2022). Tropical forests on nutrient‐rich soils tend to be more diverse, with structurally complex canopies made up of a combination of tall, light‐demanding emergent trees and an understory of shade‐tolerant species (Quesada *et al*., 2012; Jucker *et al*., 2018). The emergent species that dominate the canopy generally have resource‐acquisitive strategies that translate into faster rates of both woody productivity and mortality (Coomes *et al*., 2009; Jucker *et al*., 2016; McDowell *et al*., 2018; Muller‐Landau *et al*., 2021; Bartholomew *et al*., 2022), resulting in canopies that are highly dynamic and structurally heterogeneous. As soil nutrients become more limiting, tropical forests become increasingly dominated by species with more conservative strategies, such as shorter stature, higher wood density, slower growth, and higher survival, leading to shorter and less dynamic canopies (Quesada *et al*., 2012; Werner & Homeier, 2015; Jucker *et al*., 2018; Soong *et al*., 2020).

Similar shifts in forest composition and structure can also be observed along local topographic gradients (Chadwick & Asner, 2018, 2020; Jucker *et al*., 2018; Muscarella *et al*., 2020). Topography strongly influences soil formation and depth by affecting erosion and accretion (Vitousek *et al*., 2010; Chadwick & Asner, 2018), while also modulating exposure to wind (de Toledo *et al*., 2012; Jackson *et al*., 2021, 2024a), drought (Leitold *et al*., 2018; Miyamoto *et al*., 2021; Nunes *et al*., 2021), and waterlogging (Margrove *et al*., 2015) by shaping how air masses and water flow across landscapes. Trees growing on drier, more exposed ridges and hillslopes tend to have traits associated with greater drought resistance (Schietti *et al*., 2014; Oliveira *et al*., 2019; Esteban *et al*., 2021) and biomechanical stability (Jackson *et al*., 2024a) than those in wetter, low‐lying valleys—which in turn impacts both the structure and dynamics of the canopies they form.

But while we expect soils, topography, and water availability to constrain variation in forest structure and dynamics across tropical landscapes, quantifying their effects at these scales remains a major challenge using traditional field surveys. To address this, ecologists are increasingly turning to remote sensing technologies such as airborne laser scanning (ALS) to accurately map variation in the 3D structure of both forest canopies and the underlying terrain across whole landscapes (Kellner & Asner, 2009; Freund *et al*., 2021; Atkins *et al*., 2022; Jucker, 2022; Lines *et al*., 2022; Reis *et al*., 2022; Fischer & Jucker, 2023; LaRue *et al*., 2023). In particular, repeat‐acquisition ALS data promise to transform our ability to characterize forest canopy dynamics at landscape scales. High‐resolution canopy height models generated from sequential ALS scans can be used not only to detect and accurately measure canopy gaps and disturbances but also to monitor their subsequent recovery by tracking their evolution through time (Leitold *et al*., 2018; Dalagnol *et al*., 2021; Cushman *et al*., 2022; Csillik *et al*., 2024; Rosen *et al*., 2024; Jackson *et al*., 2024b). In doing so, ALS technologies open the door to an entirely new way of exploring the processes that govern the dynamics of tropical forests and tracking how these ecosystems are responding to rapid global change.

**Fig. 1:**
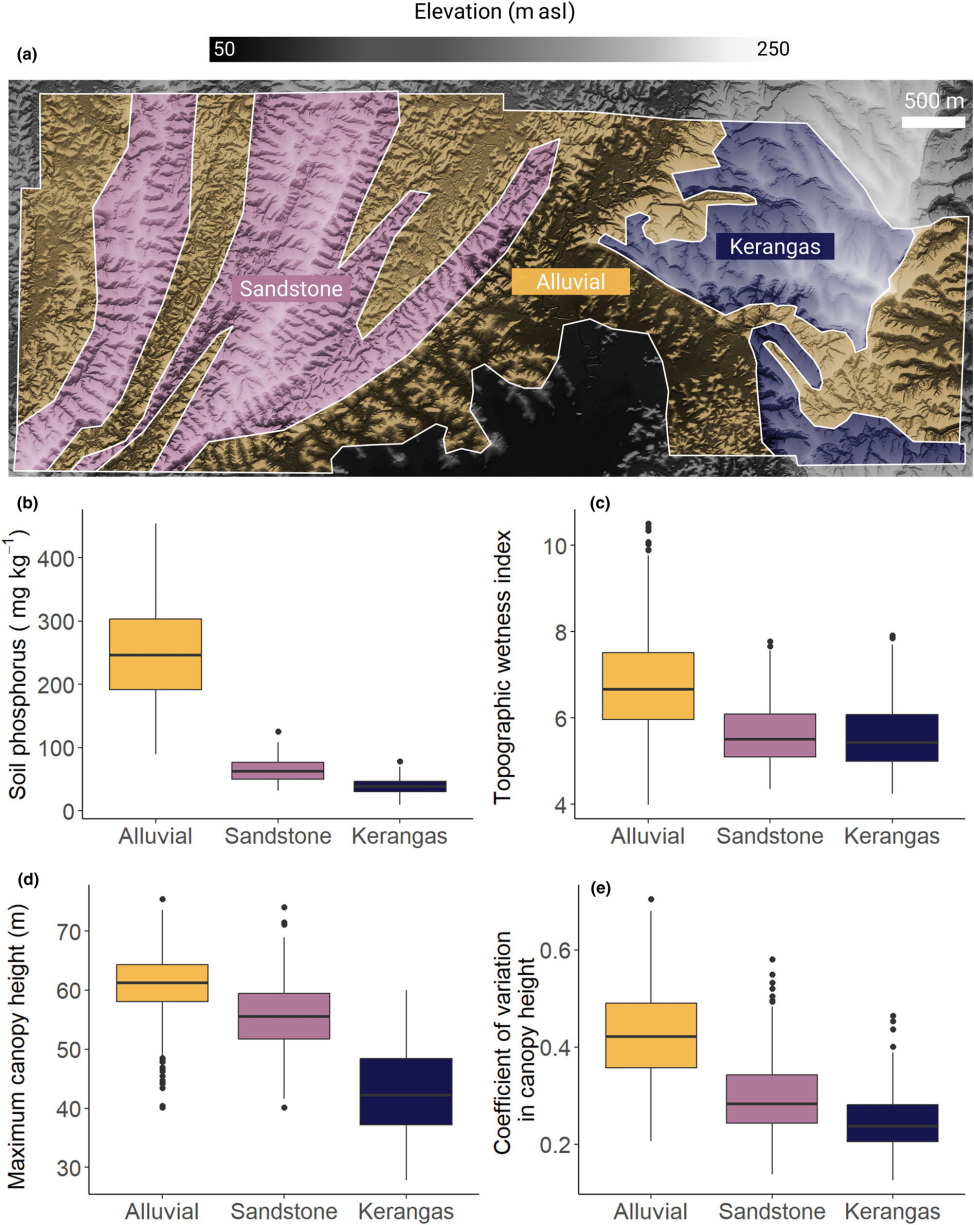
Variation in topography and forest structure across Sepilok Forest Reserve. A 1‐m resolution (a) digital terrain model (DTM) of the entire study site derived from the 2020 airborne laser scanning data is shown, with white lines outlining the study area and the boundary between the three forest types. Boxplots show the variation in (b) total soil phosphorus, (c) topographic wetness index (TWI), (d) maximum canopy height (*H*_max_), and (e) coefficient of variation in canopy height (*H*_cv_) across the three major forest types—alluvial, sandstone, and kerangas. *H*_max_, *H*_cv_, and TWI were calculated at a 1‐ha scale from the CHM and DTM (*n* = 1528 1‐ha grid cells across the landscape). Soil phosphorus was measured in the top 5 cm of soil from samples collected across nine 4‐ha plots, three for each forest type (*n* = 225 soil samples in total). Polygons outlining the distribution of the three forest types were generated based on a combination of field surveys and expert opinion, as described in Jucker *et al*. (2018).

**Fig. 2:**
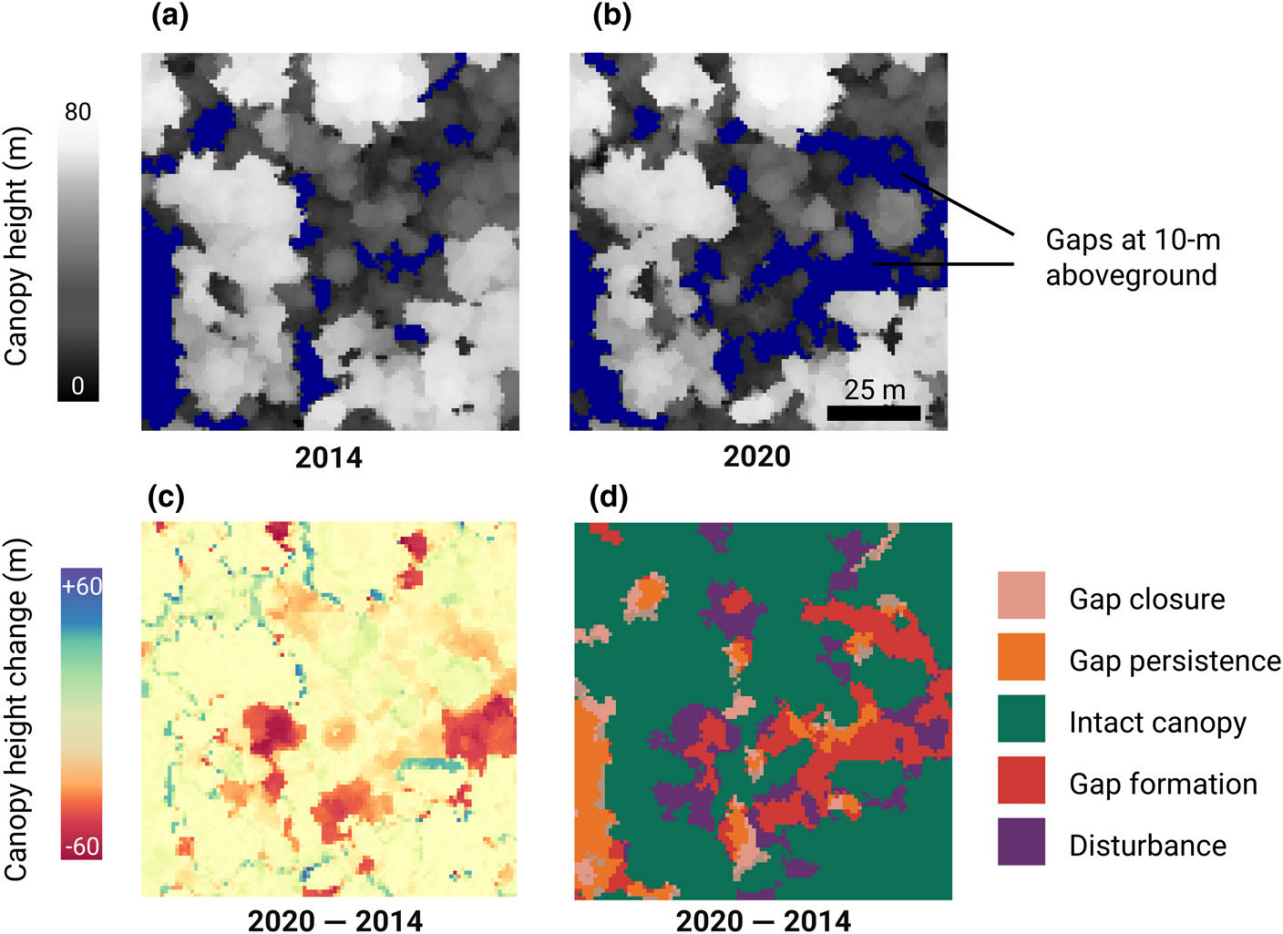
Illustration of the framework used to classify forest canopy dynamics into various components of disturbance and growth. The top row shows the canopy height model (CHM) of a 1‐ha patch of alluvial forest in (a) 2014 and (b) 2020. Areas of the canopy that meet our definition of gap (≥ 25 m^2^ and extending to within 10 m of the ground) are shown in blue. Height differences between the two CHMs highlight areas of canopy change over time (c). These were classified into five categories (d), which include areas of canopy loss (disturbances and new gap formations), gap closure, gap persistence, and intact canopy.

Here, we leverage repeat ALS data acquired across > 1500 ha of old‐growth tropical forest in Malaysian Borneo to explore how soils and topography shape the structure and dynamics of tropical forest canopies. The site is characterized by a steep gradient in soil fertility and water availability associated with local topography that gives rise to three adjacent forest types that are compositionally and structurally distinct (Dent *et al*., 2006; Jucker *et al*., 2018; Bongalov *et al*., 2019; Bartholomew *et al*., 2022), making it an ideal testbed to explore topo‐edaphic controls on canopy dynamics (Fig. 1). Using a new quantitative framework that partitions changes in canopy vertical structure into gap formation, gap closure, and intact canopy growth (Fig. 2), we tested three related hypotheses: (1) forests on low‐lying, nutrient‐rich alluvial soils experience greater rates of canopy disturbance due to the presence of taller, faster‐growing tree species that are more susceptible to disturbances such as windthrows, drought, and waterlogging; (2) higher canopy disturbance rates in alluvial forests are balanced out by faster rates of gap closure and canopy growth, reflecting a general speeding up of canopy dynamics as soil fertility increases; and (3) at the landscape scale, rates of canopy gain and loss co‐vary with forest 3D structure, with taller and more vertically heterogeneous canopies exhibiting faster canopy dynamics.

## Materials and Methods

### Study area

This study was conducted at Sepilok (5°10′N, 117°56′E), a forest reserve in the Malaysian state of Sabah in northeastern Borneo, founded in 1931 (Fox, 1973). Most of Sepilok has never been commercially logged, except for small areas in the northeast and south of the reserve that were selectively logged until 1957 (Dent *et al*., 2006). The region is characterized by a tropical climate, with a mean annual temperature of 27.6°C and annual precipitation of *c*. 3500 mm yr^−1^ (Bongalov *et al*., 2019), with occasional prolonged dry spells associated with El Niño events. The site is topographically heterogeneous, varying in elevation from *c*. 50 to 250 m above sea level (Fig. 1a), with low‐lying valleys prone to occasional flooding and waterlogging (Fig. 1c). Soil fertility decreases markedly with elevation across the site (6.4‐fold decrease in soil phosphorus from valley bottoms to ridge tops; Fig. 1b), as soils become sandier and less clayey (Bartholomew *et al*., 2022).

These differences in topography, soil fertility, and water availability give rise to three floristically and structurally distinct forest types across Sepilok (Fig. 1a): (1) alluvial dipterocarp forests in the valley bottoms; (2) sandstone dipterocarp forests on dissected hillsides and crests; and (3) kerangas forests (also known as Sundaland heath forests) that grow on nutrient‐depleted dip slopes of cuesta (Jucker *et al*., 2018). Alluvial forests are the most diverse and are home to tree species with a wide range of ecological strategies (Baltzer *et al*., 2005; Bongalov *et al*., 2019), including emergent dipterocarps that can grow 70–80 m tall (Jucker *et al*., 2018). As elevation increases, soil nutrients become progressively more limiting, and water availability decreases. These constraints act as strong ecological filters, favoring communities dominated by short‐statured species with high wood density, high nutrient‐use efficiency, increased embolism resistance, and enhanced water transport capacity (Jucker *et al*., 2018; Bartholomew *et al*., 2022; Bittencourt *et al*., 2022). Alluvial forests have the tallest canopies (Fig. 1d), but are also more vertically heterogeneous due to the presence of large canopy gaps (Fig. 1e). Sandstone forests are intermediate in terms of canopy height, but have fewer gaps and consequently also attain the highest aboveground carbon density (Coomes *et al*., 2017), while kerangas forests are considerably shorter. The main drivers of disturbance are windthrows (Jackson *et al*., 2024b), occasional droughts associated with El Niño (Qie *et al*., 2017), and waterlogging following extreme rainfall events, which mostly affect low‐lying alluvial forests (Margrove *et al*., 2015).

### Airborne laser scanning data

#### Data acquisition

Airborne laser scanning (ALS, also known as LiDAR) data were acquired across Sepilok on two occasions, first in October 2014 and again in February 2020 (5 yr and 4 months apart). The 2014 survey was conducted by NERC's Airborne Research Facility using a Leica ALS50‐II LiDAR sensor mounted on an aircraft (Jackson *et al*., 2024b). It was flown twice on the same day at altitudes of 800 and 1850 m above ground. Data from the two flights were merged, resulting in point clouds with an average density of 14 pulses m^−2^ and a scan angle of 15°. The 2020 data were collected using a helicopter flown at a lower altitude of *c*. 250 m aboveground, resulting in point clouds with an average density of 40 pulses m^−2^ and a scan angle of 30°. The point clouds were georeferenced using data from a locally operated Leica base station.

#### Data processing

All subsequent analyses were conducted in R 4.4.1 (R Core Development Team, 2024), calling LAStools to process the ALS point cloud data using the pipeline described in Fischer *et al*. (2024). This processing routine was specifically designed to generate robust and directly comparable canopy height models (CHMs) and digital terrain models (DTMs) from ALS data acquired using different airborne platforms, flight configurations, and sampling densities, and has been used in previous studies focused on multi‐site and multi‐temporal comparisons of CHMs (Fischer & Jucker, 2023; Rosen *et al*., 2024; Jackson *et al*., 2024b). Specifically, CHMs were generated using a locally adaptive spike‐free algorithm that minimizes biases arising from pulse density variation both within and between ALS surveys (Fischer *et al*., 2024; Jackson *et al*., 2024b). Both CHMs and DTMs were generated at a 1‐m resolution. Any areas of the CHMs with a density < 2 pulses m^−2^ and scan angles > 20° were masked out for all subsequent analyses, as below and above these thresholds, CHM quality degrades rapidly even when using robust algorithms (Fischer *et al*., 2024).

Even though this processing pipeline has been extensively tested across a wide range of forest types and ALS acquisitions, we nevertheless conducted an additional sensitivity analysis to assess whether our results might be affected by the approach used to reconstruct both the canopy and the underlying terrain. This revealed that our conclusions were highly robust to the choice of algorithm used to generate the CHMs, as well as potential errors arising from the ground classification (see Methods S1; Figs S4, S5).

#### Calculating topographic and canopy structural metrics

Based on existing maps that mark the distribution of the three forest types across Sepilok (Jucker *et al*., 2018), we used the 2014 CHM and 2020 DTM to calculate a series of topographic and canopy structural metrics for each forest type at 1‐ha resolution (100 × 100 m grid cells). These calculations were performed using the *terra* (Hijmans *et al*., 2024) and *whitebox* packages (Lindsay, 2016; Wu & Brown, 2024), with the DTM aggregated at 10‐m resolution to reduce noise (Jucker *et al*., 2018).

In terms of topography, we calculated the mean elevation and terrain slope of each 1‐ha grid cell, as previous work has shown these to be strong predictors of soil nutrient availability, species diversity and composition, and canopy structure at Sepilok (Jucker *et al*., 2018; Bongalov *et al*., 2019; Bartholomew *et al*., 2022). To complement this, a map of topographic wetness index (TWI) was derived at a 25‐m resolution from the resampled DTM, smoothing over small pits and mounds to better capture hillslope‐scale hydrological processes. For each 1‐ha grid cell, we then took the mean values of TWI as a measure of water availability and waterlogging risk (Margrove *et al*., 2015). High values of TWI indicate areas of the landscape where water is more likely to accumulate, which is consistent with our data showing that TWI generally decreased from alluvial to sandstone and kerangas forests (Fig. 1c). TWI and slope were strongly negatively correlated (Pearson's correlation coefficient, *r* = −0.77; Fig. S1), and as such, we did not include slope in subsequent analyses.

To characterize variation in canopy 3D structure, we calculated four metrics: (1) mean top‐of‐canopy height (*H*_mean_, mean value of all CHM pixels); (2) maximum canopy height (*H*_max_, 98% percentile of the CHM); (3) coefficient of variation in canopy height (*H*_cv_, based on the method of Lobry *et al*. (2023) which is less sensitive to outliers and is bounded between 0–1); and (4) canopy gap fraction at 10 m above ground (GF_10_, proportion of CHM pixels < 10 m). These metrics are widely used to describe different axes of canopy structure, including height, openness, and spatial heterogeneity (Atkins *et al*., 2018; Fahey *et al*., 2019; Jucker *et al*., 2023), and can be derived robustly across different sources of ALS data (Zhang *et al*., 2024). For subsequent analyses, we focused on *H*_max_ and *H*_cv_ to capture differences in canopy structure across the landscape, as these were tightly correlated with *H*_mean_ and GF_10_ (*r* = 0.59 and 0.85, respectively; Fig. S1).

Grid cells where CHM pixels with missing data made up > 5% of the 1‐ha area (e.g. due to sampling density < 2 pulses m^−2^, or the presence of water and low clouds) were excluded from the analysis. We also only retained grid cells where a single forest type made up at least 75% of the 1‐ha area, a compromise between retaining as many grid cells as possible while also limiting the inclusion of mixed pixels. This left us with a total of 1528 1‐ha grid cells, 790 in alluvial forests, 507 in sandstone forests, and 231 in kerangas forests. For a summary of how topographic and canopy structural metrics vary among forest types (see Figs 1b–e, S2; Table S1).

A 1‐ha spatial resolution was chosen on both biological and methodological grounds. Grid sizes smaller than 1‐ha are not advisable, as the dynamic processes we aim to capture (e.g. gap formation) have a minimum spatial scale that ultimately depends on the crown size of an individual canopy tree (Duncanson *et al*., 2025), which at Sepilok can exceed 35–40 m in diameter. A coarser grid would be equally problematic, as beyond 1 ha we begin to introduce substantial variability in topography and forest type within grid cells (Fig. 1a), effectively blurring the very ecological patterns we aim to detect. A grid size of 1 ha, therefore, represents a suitable compromise between these constraints. Moreover, as it is a widely used standard for tropical forest monitoring (Duncanson *et al*., 2025), it allows direct comparisons between our results and those of previous research at this and other tropical sites.

#### Calculating rates of canopy dynamics

To unpack how canopy dynamics arise from the processes of canopy disturbance, gap formation, gap closure, and canopy growth, we used a new quantitative framework proposed by Jackson *et al*. (2024b) that partitions temporal differences between CHMs into each of these components (Fig. 2). The first step was to identify canopy gaps in the 2014 and 2020 CHMs, which we did using the *ForestGapR* package (Silva *et al*., 2019). Gaps were defined as areas in the CHMs ≥ 25 m^2^ in size that extend to within 10 m or less of the ground (Fig. 2a,b). The 25 m^2^ size threshold was chosen as it is similar to the crown area of the average canopy tree. Our gap definition, therefore, encompasses individual treefalls and major crown damage, but excludes small canopy openings that can reflect spacing between tree crowns or noise in the CHMs. These fixed size and height thresholds are widely used to define gaps in ALS data acquired over tropical forests (Reis *et al*., 2022; Gorgens *et al*., 2023; Zhang *et al*., 2023), but are also arbitrary and can introduce bias when comparing structurally distinct forests (Fischer *et al*., 2024; Jackson *et al*., 2024b). We therefore tested a forest‐type specific relative height threshold (areas < 50% of mean canopy height; Dalagnol *et al*., 2021), as well as a smaller gap size threshold (≥ 10 m^2^) to account for gaps potentially being smaller in shorter forests. However, as these gave very similar results (Methods S2; Figs S6, S7), we chose to adopt the more intuitive fixed height cut‐off of 10 m aboveground for defining gaps.

Once we had mapped gaps, we then classified differences between the 2020 and 2014 CHMs into five types of dynamic processes based on changes in height (Fig. 2c,d): (1) gap formation (new gap areas that met our definition of a gap); (2) canopy disturbance (areas ≥ 25 m^2^ in size where canopy height decreased by > 5 m without extending to within 10 m of the ground and therefore not meeting our definition of gap); (3) gap persistence (areas detected as gaps at both time steps); (4) gap closure (areas initially classified as gaps in 2014 that have since recovered); and (5) intact canopy (all remaining areas that were not classified as gaps or disturbances at either timepoint). These five categories can be further grouped into canopy losses (gap formation + canopy disturbance) and canopy gains (the remaining three categories combined). Note that ‘canopy gains’ can still be negative if areas classified as either intact canopy or persistent gaps lose height between the two ALS surveys, but this only occurred in 1.7% of grid cells.

Finally, once we had generated these categorized maps of canopy dynamics, we used them to calculate three metrics of structural change for each of the 1528 1‐ha grid cells across Sepilok: (1) the proportional area covered by each of the five categories; (2) the height change (m ha^−1^ yr^−1^) in each category; and (3) the volume change (m^3^ ha^−1^ yr^−1^) in each category. Canopy volumes at both time points were calculated by multiplying canopy height by area and therefore provide an integrated measure of canopy dynamics over time.

### Variation in canopy dynamics among forest types and across the landscape

To test our first two hypotheses that canopy disturbances and growth rates vary predictably among forest types, we used ANOVAs to compare rates of canopy height and volume changes among alluvial, sandstone, and kerangas forests. Rates of canopy height and volume change were compared across the five categories of canopy dynamics (gap formation, canopy disturbance, gap persistence, gap closure, and intact canopy), and after grouping these into canopy gains, losses, and net changes (gains – losses). To aid the interpretation of results, canopy height and volume losses were converted to absolute values (i.e. the more positive the value, the greater the loss).

To test our third hypothesis that variation in rates of canopy disturbance and growth across the landscape is shaped by underlying gradients in topography and canopy structure, we fit a series of regression models relating rates of canopy volume gains, losses, and net changes to elevation, TWI, *H*_max_, and *H*_cv_. For this analysis, we focused exclusively on changes in canopy volume as measures of canopy dynamics, as they integrate the effects of canopy height and area changes. Given that the four model predictors (elevation, TWI, *H*_max_, and *H*_cv_) were themselves correlated (|*r*| = 0.27–0.71; Fig. S1), we did not attempt to compare their effect sizes in the fitted models. Instead, we assessed the ability of the models to explain variation in canopy volume gains and losses across the landscape using a leave‐one‐out spatial cross‐validation approach developed by Ploton *et al*. (2020) (see Methods S3 for details). This approach explicitly accounts for spatial autocorrelation in the gridded data, providing a much more robust estimate of the model's true predictive power—which we assessed by comparing observed and predicted values to calculate a measure of explained variance (*R* ^2^).

To compare the relative importance of topography and canopy structure in driving canopy dynamics, we fit two alternative models: one in which canopy volume gains and losses were related exclusively to elevation and TWI, and one where we also included the effects of *H*_max_ and *H*_cv_. Additionally, we also fit univariate regression models in which canopy volume gains, losses, and net changes were related to elevation, TWI, *H*_max_, and *H*_cv_ separately, while also including an interaction term with forest type (categorical variable with three levels). This allowed us to test if relationships between canopy dynamics, topography, and canopy structure observed across habitat types were also mirrored within them. Canopy volume losses (converted to absolute values) were log‐transformed before model fitting to account for their right‐skewed distribution and normalize model residuals.

## Results

### Variation in canopy structure and dynamics among forest types

Forest canopy structure varied markedly and predictably among low‐lying alluvial forests on fertile soils with higher water availability, sandstone forests on ridges, and nutrient‐depleted kerangas forests on dip slopes (Figs 1b–e, S1; Table S1). Alluvial forests had the tallest canopies (*H*_max_ = 60.8 m, mean value across 1‐ha grid cells), but also the biggest proportion of gaps (GF_10_ = 9.4%) and the greatest variability in height (*H*_cv_ = 0.43). Height decreased progressively in sandstone (*H*_max_ = 55.7 m) and kerangas forests (*H*_max_ = 43.1 m), as did gap fraction (GF_10_ = 2.0% in both forest types), with canopies becoming denser and more vertically homogeneous (*H*_cv_ = 0.30 in sandstone and 0.25 in kerangas forests).

**Fig. 3:**
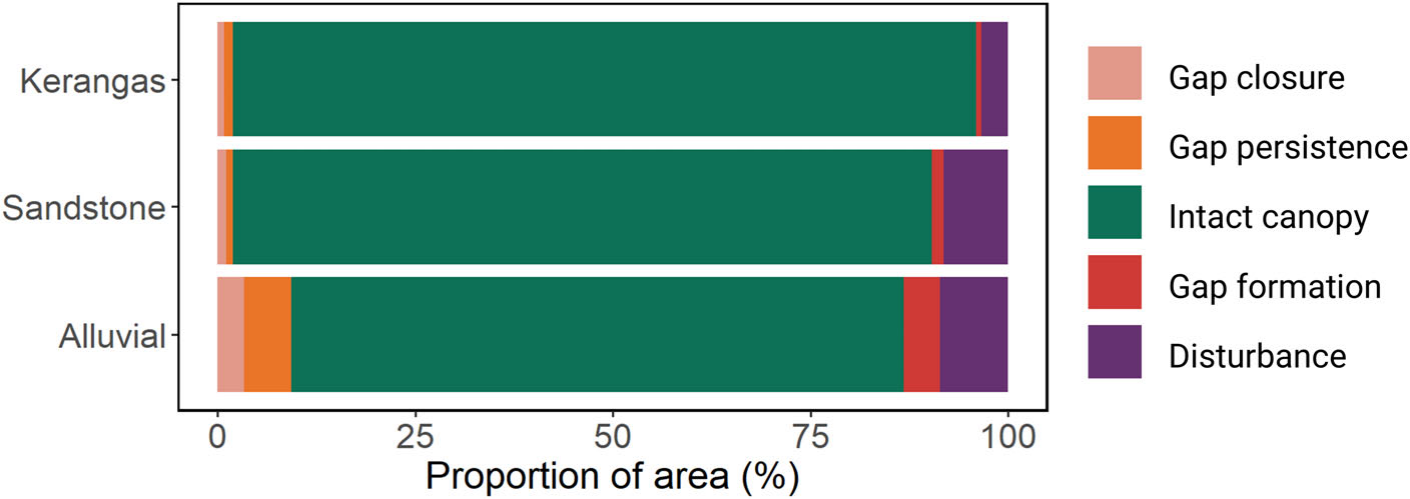
Variation in rates of canopy dynamics across the three forest types. Each bar corresponds to the proportion of the canopy area that either remained intact between 2014 and 2020 (green), was classified as a gap at both time steps (orange), underwent canopy closure (pink), or was subjected to a new disturbance (red and purple). For a description of the five classes of canopy dynamics, see the main text and Fig. 2.

**Fig. 4:**
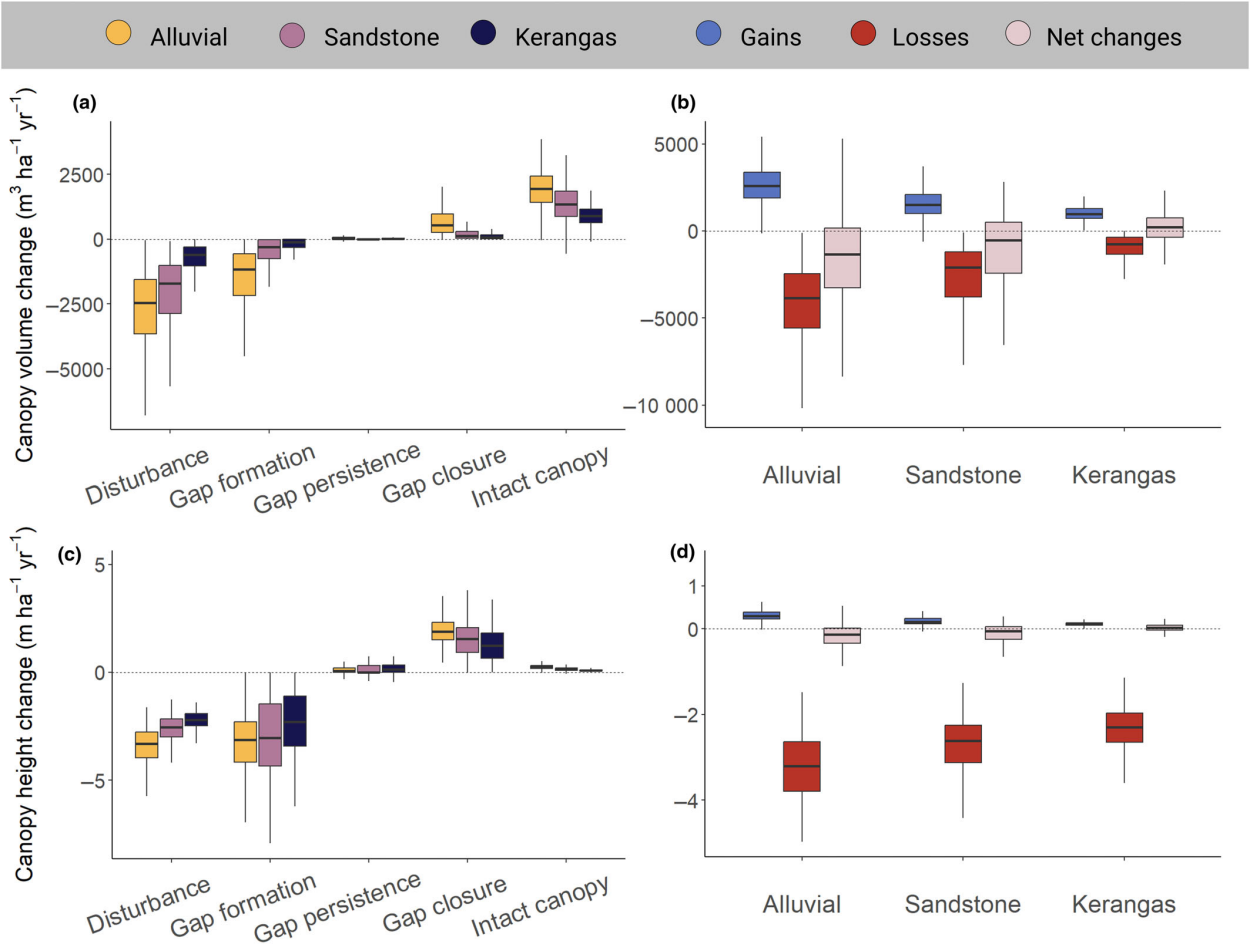
Variation in canopy volume and height change across the three forest types. Boxplots on the top row show variation in volume change, both (a) across the five canopy classes of canopy dynamics and (b) aggregated into canopy gains, losses, and net changes. The bottom row (c and d) shows the same results for canopy height change. See Supporting Information Table S1 for pairwise comparisons between forest types based on ANOVAs. For a description of the five classes of canopy dynamics, see the main text and Fig. 2. Outliers are not shown on the boxplots.

Canopy dynamics also varied considerably among forest types (Figs 3, 4; Table S1). Across Sepilok, most of the landscape was classified as ‘intact canopy’ (77–94% across forest types; green bars in Fig. 3). However, the proportion of the canopy area that was either recovering from previous disturbances or affected by new ones that occurred between the two ALS surveys differed clearly between forest types. The proportion of canopy area that was affected by disturbance was noticeably higher in alluvial forests (13%; red and purple bars in Fig. 3) than in sandstone (10%) and kerangas forests (4%). Similarly, alluvial forests had the highest proportion of canopy area that underwent recovery between 2014 and 2020 (3%; pink bar in Fig. 3).

As predicted, the combination of taller canopies and higher disturbance risk meant that rates of canopy volume loss were significantly greater in alluvial forests (4674 m^3^ ha^−1^ yr^−1^; Fig. 4b; Table S1) than in sandstone (2687 m^3^ ha^−1^ yr^−1^) and kerangas forests (1005 m^3^ ha^−1^ yr^−1^). At the same time, gains in canopy volume also increased progressively from kerangas (1054 m^3^ ha^−1^ yr^−1^) to sandstone (1565 m^3^ ha^−1^ yr^−1^) and alluvial forests (2651 m^3^ ha^−1^ yr^−1^), partially or completely balancing out losses. Canopy height increases were generally fastest in gaps that closed between 2014 and 2020 and peaked in alluvial forests (2 m ha^−1^ yr^−1^; Fig. 4c). Areas of intact canopy also generally increased in height, but at a much slower rate (Fig. 4c), with alluvial forests once again exhibiting faster rates of canopy growth (0.3 m ha^−1^ yr^−1^) than sandstone (0.2 m ha^−1^ yr^−1^) and heath forests (0.1 m ha^−1^ yr^−1^). However, because intact canopies make up the majority of the landscape in terms of area (Fig. 3), when height increments were converted into volume gains, we found that most of the canopy volume increases were driven by growth of the intact canopy rather than recovery within gaps (Fig. 4a).

On balance across the whole landscape, canopy gains resulting from gap closure and mature tree growth only partially offset losses driven by disturbances (Fig. 4b,d). In particular, both alluvial and sandstone forests were shorter on average (net change in height = −0.2 m ha^−1^ yr^−1^ and –0.1 m ha^−1^ yr^−1^, respectively) and had lower total canopy volume in 2020 than in 2014 (net change in volume = −2023 m^3^ ha^−1^ yr^−1^ and –1122 m^3^ ha^−1^ yr^−1^, respectively). By contrast, kerangas forests not only had the lowest rates of canopy dynamics, but overall gained slightly more canopy than they lost over the study period (net change in height = +0.01 m ha^−1^ yr^−1^; net change in volume = +48 m^3^ ha^−1^ yr^−1^).

### Shifts in canopy dynamics along gradients of topography and canopy structure

**Fig. 5:**
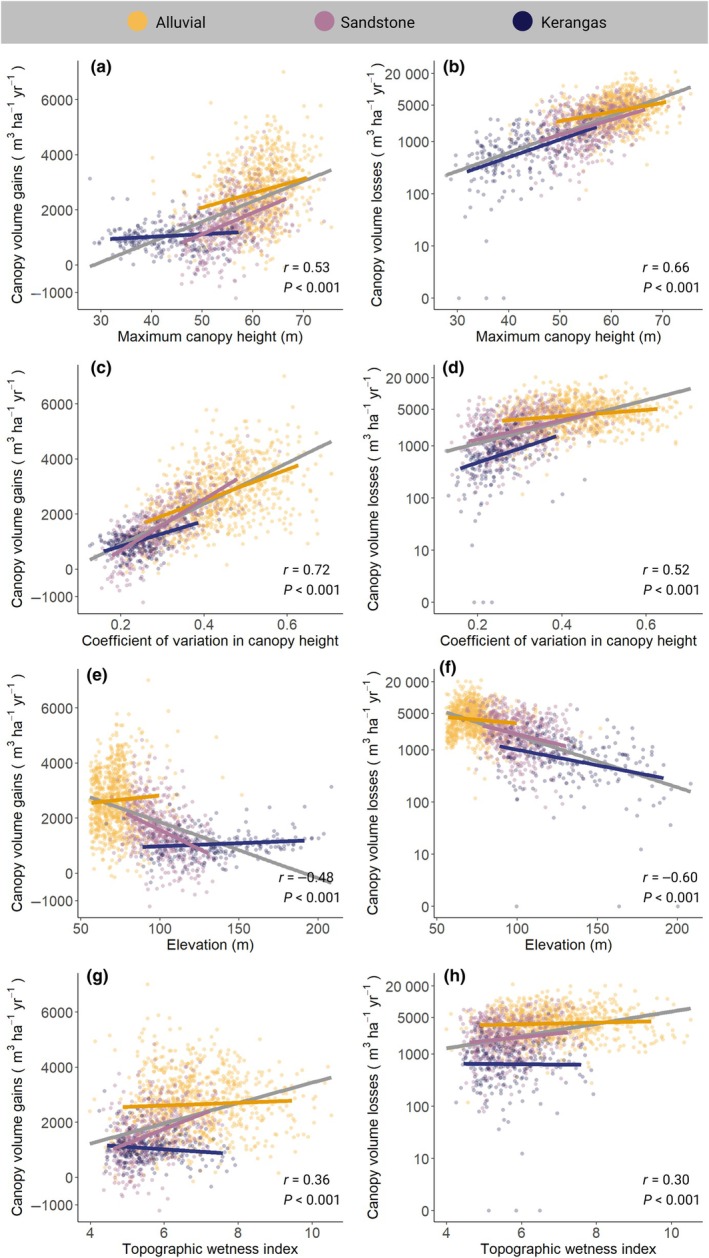
Variation in canopy volume gains and losses across Sepilok Forest Reserve in relation to topography and canopy 3D structure (a–h). Each point represents a 1‐ha plot, while lines show the fit of univariate regression models with an interaction term with forest type. Gray lines show the fit across the entire dataset, with corresponding *P*‐values and Pearson's correlation coefficients (*r*) for each bivariate relationship reported at the bottom of the panels. Colored lines illustrate how relationships vary among forest types. See Supporting Information Table S2 for a summary of model fit statistics.

Topography and canopy 3D structure emerged as strong predictors of variation in rates of canopy volume gains and losses across the landscape (Fig. 5; Table S2). Overall, the greatest rates of canopy turnover were experienced by taller forests with more vertically heterogeneous canopies that grow at lower elevations within the landscape, where soils are more fertile and more prone to waterlogging. In particular, both canopy volume gains and losses decreased with elevation (*r* = −0.48 and −0.60, respectively) and increased with TWI across the landscape (*r* = 0.36 and 0.30, respectively). Similarly, canopy volume gains and losses both increased markedly with *H*_max_ (*r* = 0.53 and 0.66, respectively) and *H*_cv_ (*r* = 0.72 and 0.52, respectively). These consistent patterns emerged despite the fact that canopy volume gains and losses are themselves only weakly positively correlated across the landscape (*r* = 0.27), indicating that they mostly vary independently of one another at the 1‐ha scale. When gains and losses were combined into net changes in canopy volume, we found that these became increasingly negative in taller alluvial forests near valley bottoms (Fig. S3).

Multiple regression models that included the effects of both topography and canopy structure explained 53% of the variation in canopy volume gains and 44% of the variation in losses at the 1‐ha scale. This was noticeably higher than models that only included the effects of elevation and TWI (*R* ^2^ = 0.28 and 0.37 for canopy volume gains and losses, respectively), indicating that both topography and canopy structure contribute to driving landscape‐scale variation in canopy dynamics. Overall, univariate models relating canopy volume gains and losses to elevation, TWI, *H*_max_, and *H*_cv_ separately revealed that, in most cases, patterns observed across the entire landscape were also mirrored within forest types (Fig. 5). The one exception was TWI, where we found little variation in rates of canopy dynamics along wetness gradients within forest types (Fig. 5g,h).

## Discussion

### Soils and topography underpin large differences in canopy dynamics among forest types

Our study revealed that soils and topography not only shape the 3D structure and composition of tropical forest canopies (Quesada *et al*., 2012; Chadwick & Asner, 2018; Jucker *et al*., 2018; Bongalov *et al*., 2019; Muller‐Landau *et al*., 2021), but also profoundly alter their dynamics. Previous work has found that canopy disturbances—which, despite being relatively rare, are disproportionately important for driving forest dynamics (Johnson *et al*., 2016; Pugh *et al*., 2019a; Muller‐Landau *et al*., 2021)—are influenced by soil properties and subtle terrain features in a Panamanian tropical forest (Cushman *et al*., 2022). However, our study is the first to show that soils and topography also constrain rates of canopy recovery and growth across the landscape, both directly and through their influence on canopy 3D structure.

Canopy openings caused by treefalls and large branchfalls were much more frequent and larger in low‐lying alluvial forests growing on fertile soils. This is consistent with previous observations at Sepilok showing that infrequent extreme rainfall events predominantly impact alluvial forests through waterlogging (Margrove *et al*., 2015). Similarly, tall, emergent dipterocarps that have grown in sheltered alluvial valleys are likely to be more susceptible to windthrows during rare storm events. This vulnerability is a result of their physical exposure to wind (e.g. large, emergent crowns) and their tendency to prioritize height growth over biomechanical stability (e.g. low wood density, high stem slenderness; Coomes *et al*., 2018, Jackson *et al*., 2024a). Additionally, some of the disturbance events we detected across Sepilok likely reflect the legacy of the strong 2015–16 El Niño drought, which, like previous El Niño events, led to increased tree mortality across Sabah (Qie *et al*., 2017; Miyamoto *et al*., 2021). Given the greater susceptibility to drought of tall, fast‐growing species such as those that dominate the canopy of alluvial forests (Tavares *et al*., 2023; Chen *et al*., 2024), it is conceivable that this period of drought contributed to the high rates of canopy loss observed in alluvial forests. However, previous work has shown that during the 2015–16 El Niño, tree mortality was particularly pronounced in forests growing on exposed ridges (Nunes *et al*., 2021), with similar increases in mortality reported in both kerangas and lowland dipterocarp forests (Miyamoto *et al*., 2021). Consequently, drought is likely to be but one of several drivers behind the spatial patterns in canopy disturbances we observe across Sepilok.

Higher rates of disturbance in wet and fertile valleys were also accompanied by much faster rates of gap recovery and canopy growth. Alluvial forests are home to a wide range of tall, fast‐growing dipterocarp species that can rapidly exploit gap openings both from below and through lateral crown expansion (Jucker *et al*., 2018; Bartholomew *et al*., 2022). By contrast, species on sandstone ridges and, in particular, those in drier and nutrient‐depleted kerangas forests tend to adopt much more conservative ecological strategies (Margrove *et al*., 2015; Bartholomew *et al*., 2022), making them less prone to disturbance but also slower growing (Ordway *et al*., 2022).

### Predictable shifts in canopy gains and losses across tropical forest landscapes

Our study also revealed that a substantial amount of the variation in canopy dynamics across the landscape can be explained by the 3D structure of the canopy and the underlying terrain. Specifically, we found that taller and more vertically heterogeneous forest patches growing in valley bottoms where water readily accumulates had both the highest rates of canopy volume losses and gains. This is consistent with previous research showing that tropical forests on fertile soils in alluvial valleys—that tend to be dominated by fast‐growing, light‐demanding species—are not only taller and more structurally complex, but also more dynamic and prone to disturbances (Quesada *et al*., 2012; Jucker *et al*., 2018; Muller‐Landau *et al*., 2021; Cushman *et al*., 2022). Gains in canopy volume were generally more predictable than losses (*R* ^2^ = 0.53 vs 0.44; Table S2), which is intuitive as the former are a result of a gradual process of accrual, while disturbances are much more stochastic in nature (Fisher *et al*., 2008; Chambers *et al*., 2013). Coarsening the resolution of the analysis (e.g. by increasing grid cell size from 1 to 10–50 ha) would likely increase the predictability of disturbance rates by integrating sporadic events over a larger forest area (Cushman *et al*., 2022).

Our results highlight the transformative role that technologies such as ALS can play in allowing us to better quantify and map forest canopy dynamics at scale. This newfound understanding of the processes that govern canopy gains and losses will prove critical to building the next generation of forest dynamics models (Fischer *et al*., 2019; Pugh *et al*., 2019a,b; Jucker, 2022), as well as training and validating global observatories of forest aboveground biomass stocks and fluxes from satellites (Saatchi *et al*., 2011; Schimel *et al*., 2015; Asner *et al*., 2018; Harris *et al*., 2021). In particular, they will help identify where satellite‐derived maps of forest height (Lang *et al*., 2023; Tolan *et al*., 2024) fail to capture variation in forest structure and dynamics within landscapes (Moudrý *et al*., 2024). Not only do these products systematically underestimate height (and therefore aboveground biomass) in tall, closed‐canopy forests (Moudrý *et al*., 2024), but their coarser native resolution (10–30 m) also misses local‐scale heterogeneity in canopy structure captured by ALS.

However, in this context, one important distinction to make is between what ALS actually measures (canopy 3D structure and space filling) and what these data are used to infer (aboveground biomass). For example, the high rates of canopy growth observed in alluvial forests, especially those in gaps, are driven by a combination of vertical tree growth and lateral crown expansion (Krüger *et al*., 2024). In these tall forests, even small increases in the crown extent of emergent trees can drive big increases in mean canopy height (see Methods S4; Figs S8–S10 for details; Fig. 2c for a visual example). But while lateral crown expansion may profoundly impact the surface of the outer canopy captured by the CHM, it will have only a minor impact on forest biomass stocks, as most of the tree's aboveground biomass is stored in its stem and major branches (Chave *et al*., 2014). This reflects an essential uncertainty that should be carefully considered when using ALS to convert canopy 3D structural information into estimates of forest biomass stocks (Mascaro *et al*., 2011) and fluxes (Leitold *et al*., 2018).

### Tropical forests out of ‘*sink*’?

When we added up canopy volume gains and losses across the Sepilok landscape between 2014 and 2020, we found that, on average, more canopy was lost than gained. Given the relatively short timeframe of our study, it is hard to say whether or not this is an indication of a system shifting away from its historical steady‐state baseline (Chambers *et al*., 2013). Our robust processing pipeline and previous analyses rule out strong biases linked to differences in the two ALS datasets (Fischer *et al*., 2024; Jackson *et al*., 2024b). Instead, our findings are consistent with reports from plot data across the tropics, indicating that tree mortality rates are on the rise and are not being offset by increases in woody productivity linked to CO_2_ fertilization (Qie *et al*., 2017; Hubau *et al*., 2020; McDowell *et al*., 2020; Bauman *et al*., 2022; Csillik *et al*., 2024; Pan *et al*., 2024). In particular, we observed that tall, fast‐growing alluvial forests were those that experienced the greatest net losses in canopy volume, whereas kerangas forests dominated by more ecologically conservative species actually accrued a small amount of canopy volume over the same period. This could indicate that tall alluvial forests are becoming increasingly susceptible to climate extremes, such as droughts associated with the 2015–16 El Niño (Bennett *et al*., 2015; Qie *et al*., 2017; Stovall *et al*., 2019). But it could also simply be a reflection of the ‘rapid out, slow in’ nature of forest dynamics (Körner, 2003; Chambers *et al*., 2013), where episodic disturbances that cause large canopy losses are followed by protracted periods of gradual recovery that unfold over decades (Poorter *et al*., 2016; Cook‐Patton *et al*., 2020).

To better understand how old‐growth tropical forests in Borneo and elsewhere are being impacted by rapid global change, large‐scale and long‐term monitoring approaches that integrate both field and remote sensing data are essential. While several well‐coordinated forest ground monitoring networks already exist (e.g. ForestGEO and ForestPlots), it is only in recent years that we have begun to see parallel initiatives to bring together and harmonize ALS data acquired over forests (Duncanson *et al*., 2022; Jucker, 2022; Labrière *et al*., 2023). It is through international collaborations such as GEO‐TREES (https://geo‐trees.org) and infrastructure investments like NEON in the US (https://www.neonscience.org) and TERN in Australia (https://www.tern.org.au) that we can begin to build a detailed picture of how the world's forests are responding and adapting to climate change.

## Competing interests

None declared.

## Author contributions

BZ and TJ designed the study, with assistance from TDJ. DAC and TDJ led the acquisition of the airborne laser scanning data, with assistance from TJ and DFRPB. DFRPB and RN coordinated the network of permanent forest plots at Sepilok Forest Reserve, with assistance from PRLB, DCB and LR. BZ led the processing and analysis of the data, with assistance from TJ, TDJ and FJF. BZ wrote the first draft of the manuscript, with assistance from TJ, and all other authors contributed substantially to revisions.

## Disclaimer

The New Phytologist Foundation remains neutral with regard to jurisdictional claims in maps and in any institutional affiliations.

## Supporting information


**Fig. S1** Covariation among topographic and canopy structural metrics.
**Fig. S2** Variation in topography and canopy structure among forest types.
**Fig. S3** Net changes in canopy volume.
**Fig. S4** Variation in canopy dynamics rates using alternative CHM algorithm and ground classification.
**Fig. S5** Variation in canopy height and volume change using alternative CHM algorithm and ground classification.
**Fig. S6** Variation in canopy dynamics rates using alternative gap and disturbance definitions.
**Fig. S7** Variation in canopy height and volume change using alternative gap and disturbance definitions.
**Fig. S8** A framework for classifying forest canopy dynamics incorporating lateral crown expansion.
**Fig. S9** Variation in canopy dynamics rates incorporating lateral crown expansion.
**Fig. S10** Variation in canopy height and volume change incorporating lateral crown expansion.
**Methods S1** Sensitivity of canopy dynamics to CHM algorithm and ground classification.
**Methods S2** Sensitivity of canopy dynamics to gap definition.
**Methods S3** Spatial cross‐validation of regression models.
**Methods S4** Drivers of gap closure: vertical growth and lateral crown expansion.
**Table S1** Differences in canopy structure and dynamics among forest types.
**Table S2** Summary of regression models of canopy volume dynamics.
Please note: Wiley is not responsible for the content or functionality of any Supporting Information supplied by the authors. Any queries (other than missing material) should be directed to the *New Phytologist* Central Office.

## Data Availability

All data and R code needed to replicate the results of this study are publicly available on Zenodo (doi: 10.5281/zenodo.15547937). The airborne laser scanning data acquired in both 2014 and 2020 are permanently archived on Zenodo (doi: 10.5281/zenodo.10908679) and NERC's Centre for Environmental Data Analysis (CEDA) archive (https://data.ceda.ac.uk/neodc/arsf/2014/MA14_14 and doi: 10.5285/dd4d20c8626f4b9d99bc14358b1b50fe).
