# Supplementary material for: Soils and topography drive large and predictable shifts in canopy dynamics across tropical forest landscapes
Source: New Phytol. 2025 Jun 13;247(4):1666–79. doi: 10.1111/nph.70300 (PMC12267913; doi:10.1111/nph.70300)
Supplement: Supplementary file 1 — Fig. S1 Covariation among topographic and canopy structural metrics. Fig. S2 Variation in topography and canopy structure among forest types. Fig. S3 Net changes in canopy volume. Fig. S4 Variation in canopy dynamics rates using alternative CHM algorithm and ground classification. Fig. S5 Variation in canopy height and volume change using alternative CHM algorithm and ground classification. Fig. S6 Variation in canopy dynamics rates using alternative gap and disturbance definitions. Fig. S7 Variation in canopy height and volume change using alternative gap and disturbance definitions. Fig. S8 A framework for classifying forest canopy dynamics incorporating lateral crown expansion. Fig. S9 Variation in canopy dynamics rates incorporating lateral crown expansion. Fig. S10 Variation in canopy height and volume change incorporating lateral crown expansion. Methods S1 Sensitivity of canopy dynamics to CHM algorithm and ground classification. Methods S2 Sensitivity of canopy dynamics to gap definition. Methods S3 Spatial cross‐validation of regression models. Methods S4 Drivers of gap closure: vertical growth and lateral crown expansion. Table S1 Differences in canopy structure and dynamics among forest types. Table S2 Summary of regression models of canopy volume dynamics. Please note: Wiley is not responsible for the content or functionality of any Supporting Information supplied by the authors. Any queries (other than missing material) should be directed to the New Phytologist Central Office. [file NPH-247-1666-s001.pdf]

New Phytologist

New Phytologist Supporting Information

Article title: Soils and topography drive large and predictable shifts in canopy dynamics across tropical forest landscapes

Authors: Beibei Zhang, Toby D. Jackson, David A. Coomes, David F.R.P. Burslem, Reuben Nilus, Paulo R. L. Bittencourt, David C. Bartholomew, Lucy Rowland, Fabian J. Fischer and Tommaso Jucker

Article acceptance date: 27 May 2025

The following Supporting Information is available for this article:

Fig. S1 Covariation among topographic and canopy structural metrics

Fig. S2 Variation in topography and canopy structure among forest types

Fig. S3 Net changes in canopy volume

Fig. S4 Variation in canopy dynamics rates using alternative CHM algorithm and ground classification

Fig. S5 Variation in canopy height and volume change using alternative CHM algorithm and ground classification

Fig. S6 Variation in canopy dynamics rates using alternative gap and disturbance definitions

Fig. S7 Variation in canopy height and volume change using alternative gap and disturbance definitions

Fig. S8 A framework for classifying forest canopy dynamics incorporating lateral crown expansion

Fig. S9 Variation in canopy dynamics rates incorporating lateral crown expansion

Fig. S10 Variation in canopy height and volume change incorporating lateral crown expansion

Methods S1 Sensitivity of canopy dynamics to CHM algorithm and ground classification

Methods S2 Sensitivity of canopy dynamics to gap definition

Methods S3 Spatial cross-validation of regression models

Methods S4 Drivers of gap closure: vertical growth and lateral crown expansion

Table S1 Differences in canopy structure and dynamics among forest types

Table S2 Summary of regression models of canopy volume dynamics

Fig. S1 – Covariation among topographic and canopy structural metrics

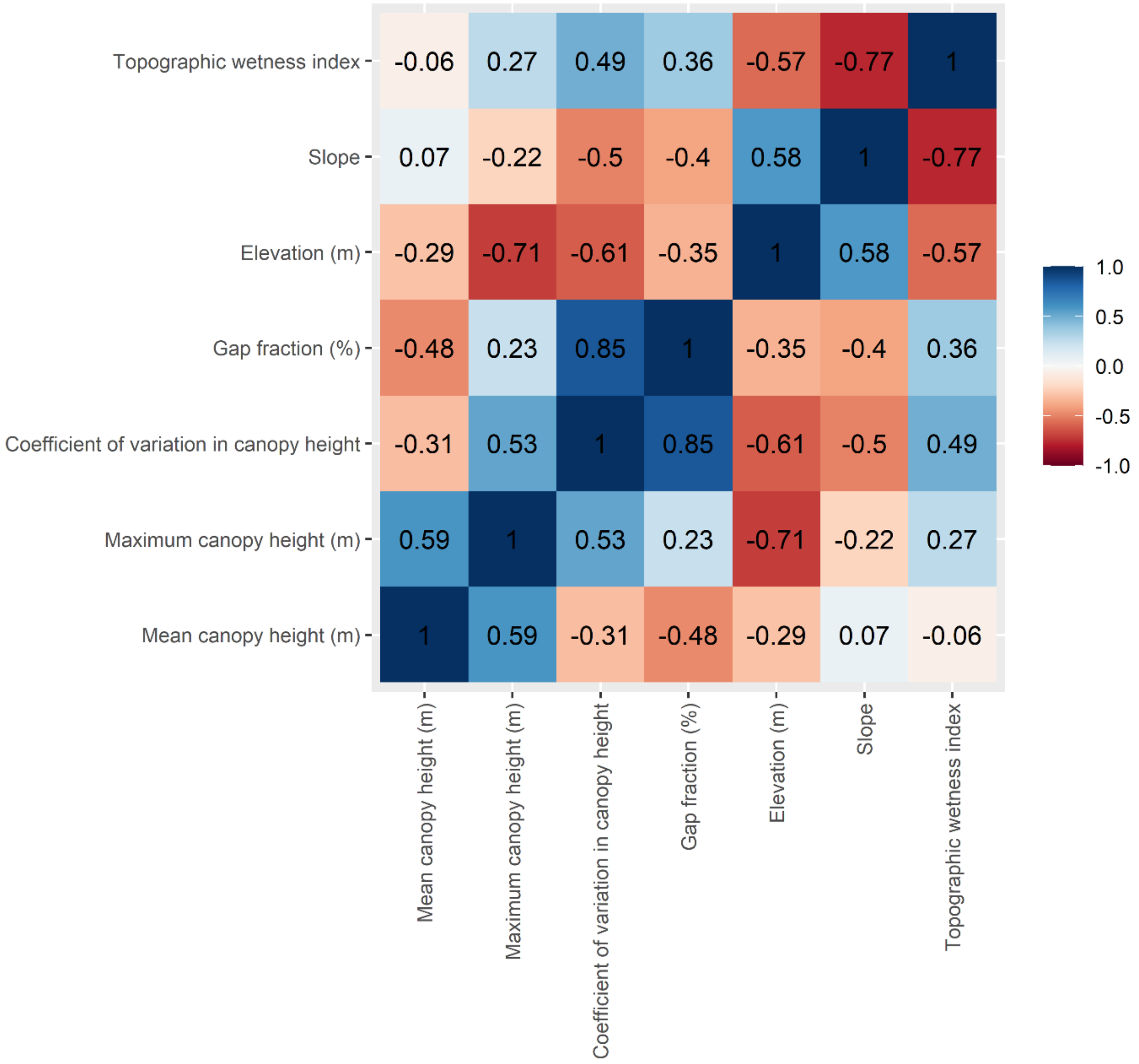

Fig. S1: Correlation heatmap among topographic and canopy structural metrics. Pearson's correlation coefficients for each pairwise relationship is reported.

Fig. S2 – Variation in topography and canopy structure among forest types

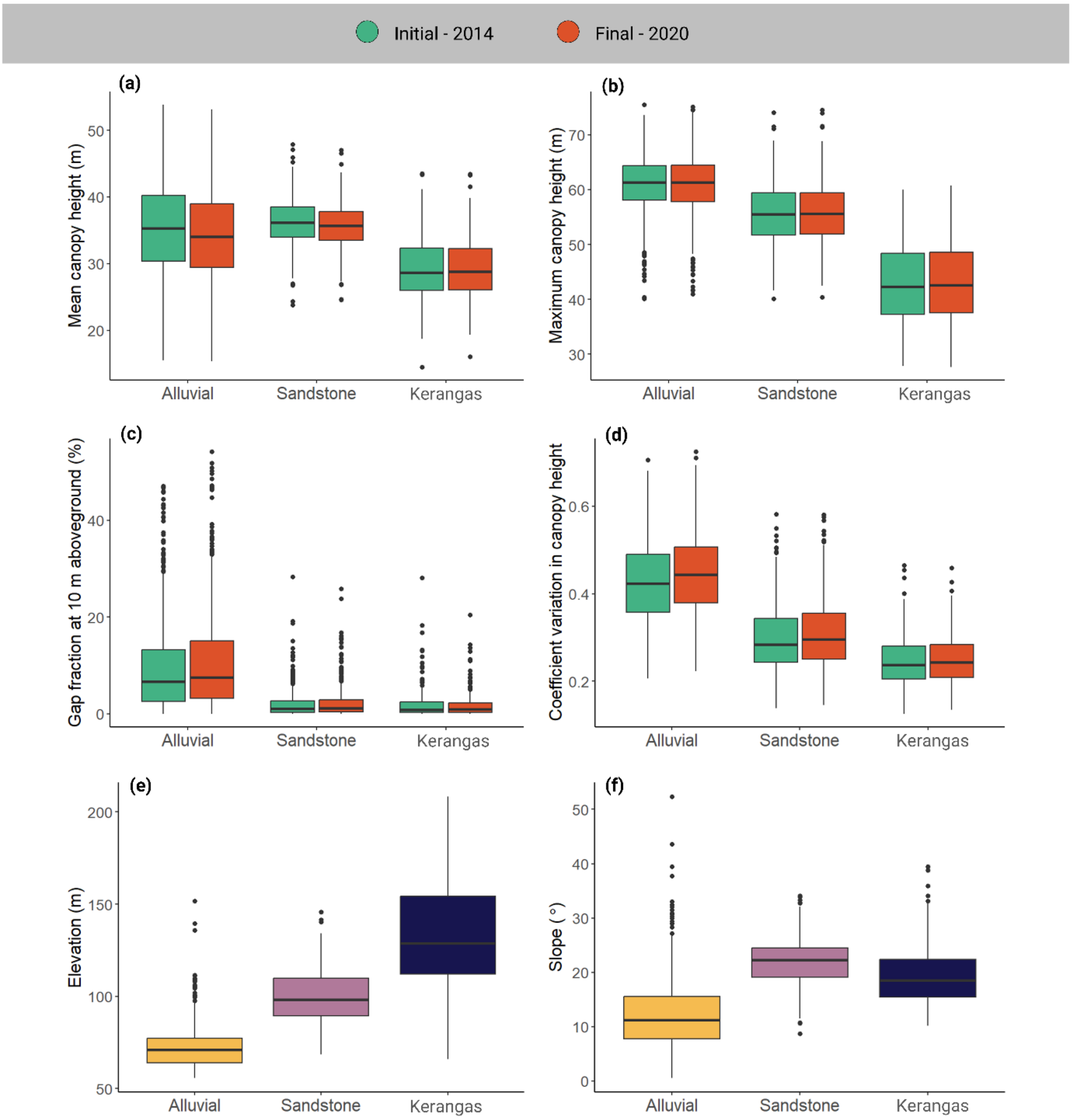

Fig. S2: Variation in canopy structural (**a–d**) and topographic metrics (**e–f**) among the three forest types. For the canopy structural metrics, differences are shown for both the 2014 (green) and 2020 (red) ALS scans.

Fig. S3 – Net changes in canopy volume

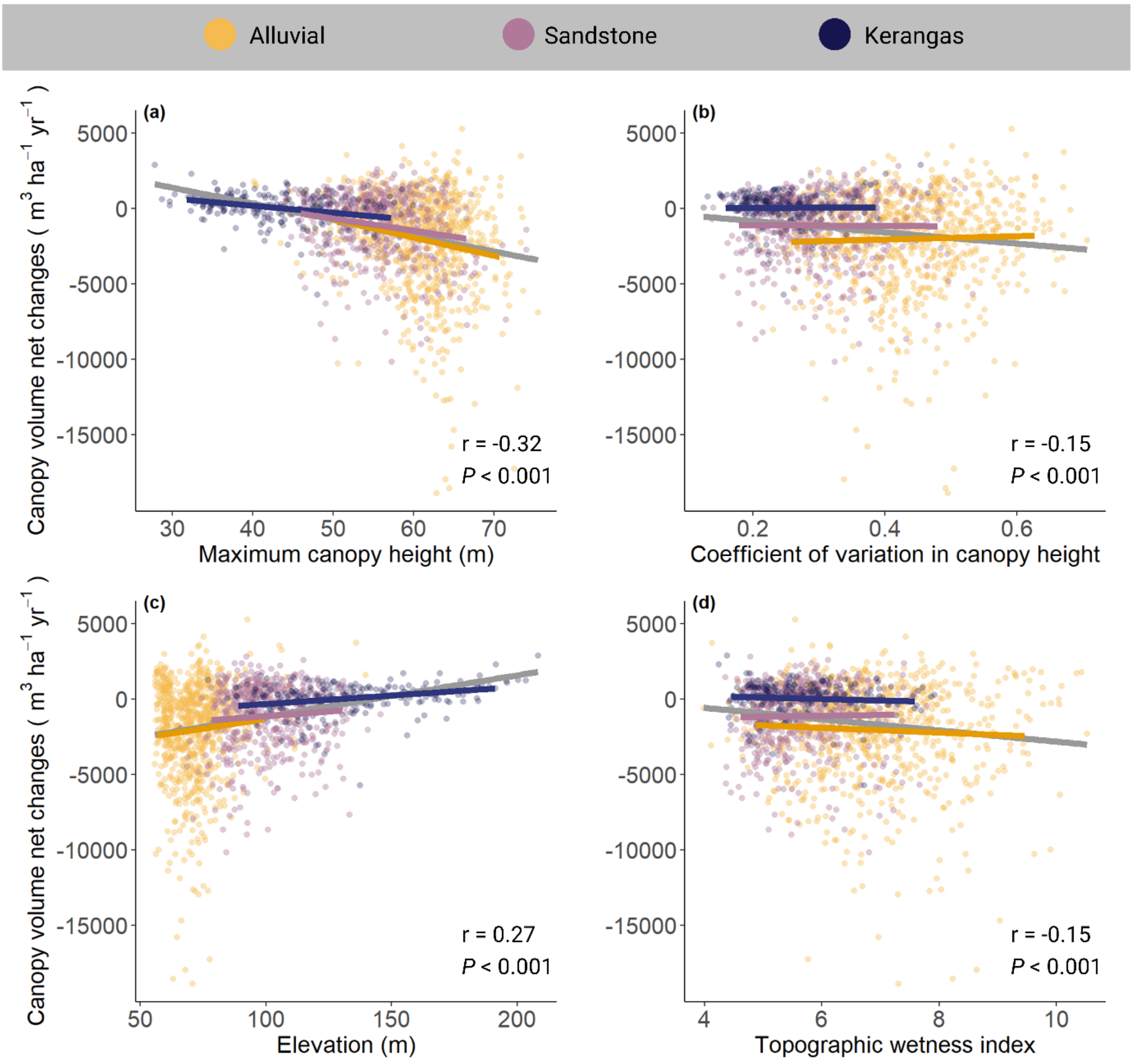

Fig. S3: Variation in net changes in canopy volume across Sepilok Forest Reserve in relation to topography and canopy structure. Net changes in canopy volume were calculated as volume gains – losses, so that values >0 indicate net volume gains while those <0 correspond to net losses. Each point represents a 1-ha plot, while lines show the fit of univariate regression models with an interaction term with forest type. Grey lines show the fit across the entire dataset, with corresponding *P*-values and Pearson's correlation coefficients (*r*) for each bivariate relationship reported in the bottom right side of the panels. Coloured lines illustrate how these relationships vary among forest types.

Fig. S4 – Variation in canopy dynamics rates using alternative CHM algorithm and ground classification

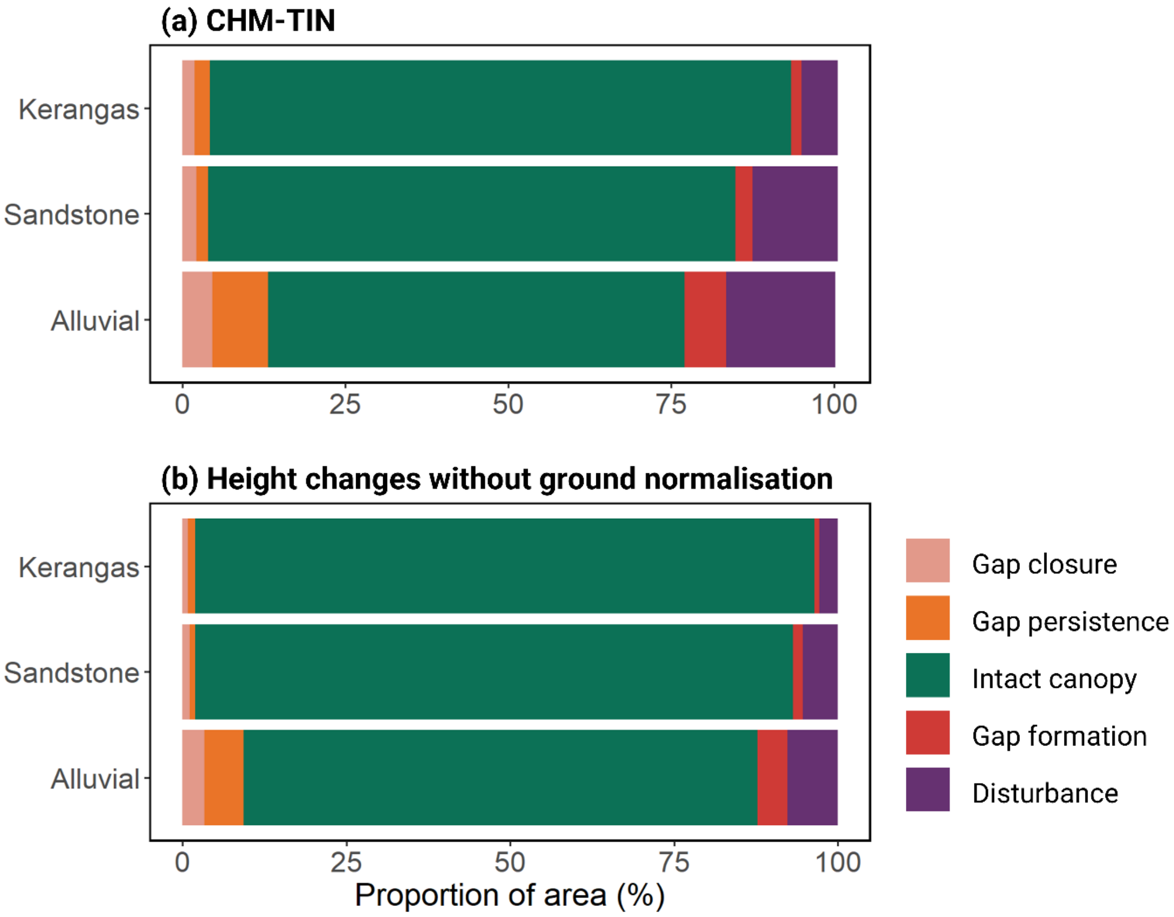

Fig. S4: Variation in rates of canopy dynamics across the three forest types using (**a**) CHMs generated using the TIN algorithm and (**b**) based on height changes inferred directly from the DSMs without normalising these relative to ground heights from DTMs. Each bar corresponds to the proportion of the canopy area that either remained intact between 2014 and 2020 (green), was classified as a gap at both time steps (orange), underwent canopy closure (pink), or was subjected to a new disturbance (red and purple). This figure is analogous to Fig. 3 in the main text.

Fig. S5 – Variation in canopy height and volume change using alternative CHM algorithm and ground classification

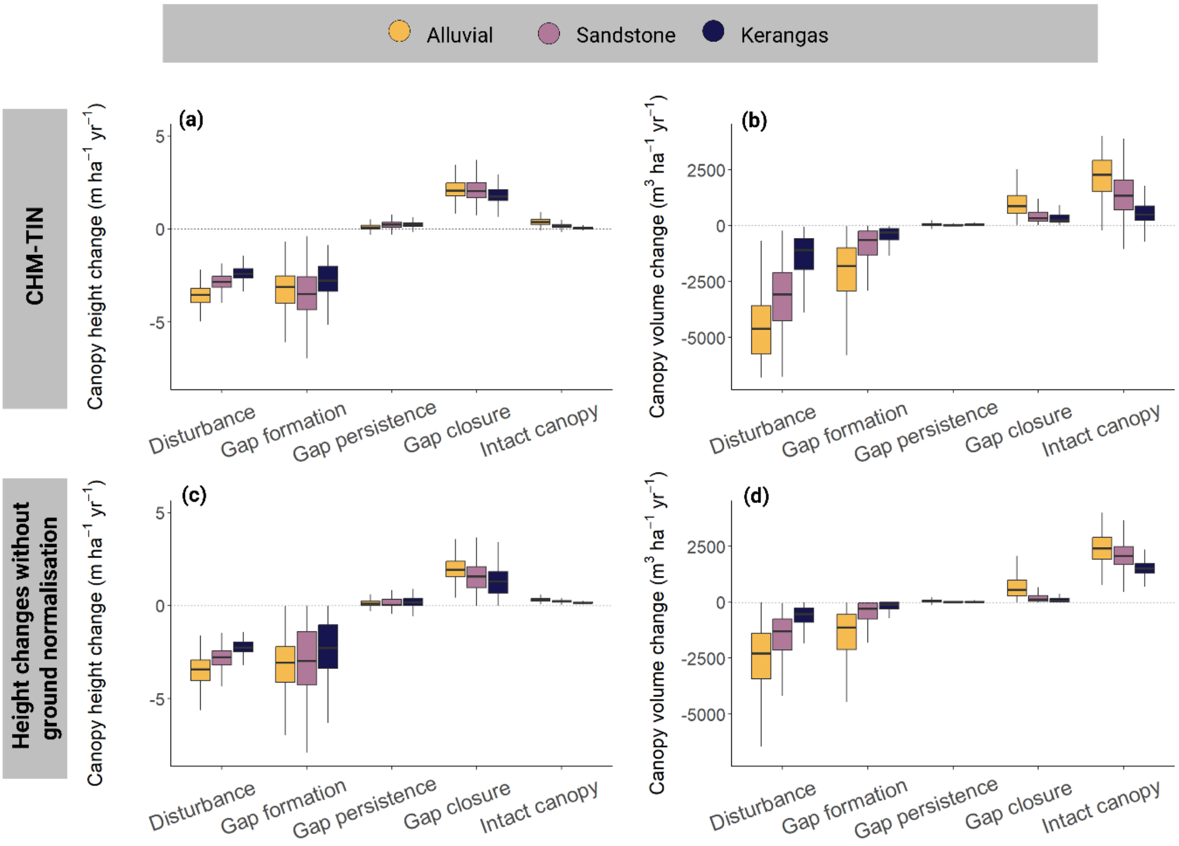

Fig. S5: Variation in canopy height and volume change across the three forest types using (**a–b**) CHMs generated using the TIN algorithm and (**c–d**) based on height changes inferred directly from the DSMs without normalising these relative to ground heights from DTMs. This figure is analogous to Fig. 4 in the main text.

Fig. S6 – Variation in canopy dynamics rates using alternative gap and disturbance definitions

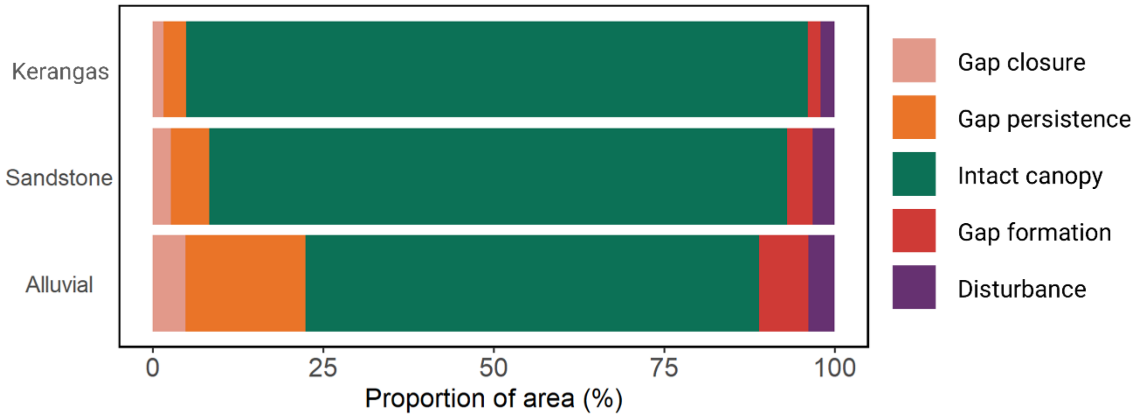

Fig. S6: Variation in rates of canopy dynamics across the three forest types using the alternative definition of canopy gaps and disturbances described in Methods S2. Each bar corresponds to the proportion of the canopy area that either remained intact between 2014 and 2020 (green), was classified as a gap at both time steps (orange), underwent canopy closure (pink), or was subjected to a new disturbance (red and purple). This figure is analogous to Fig. 3 in the main text.

Fig. S7 – Variation in canopy height and volume change using alternative gap and disturbance definitions

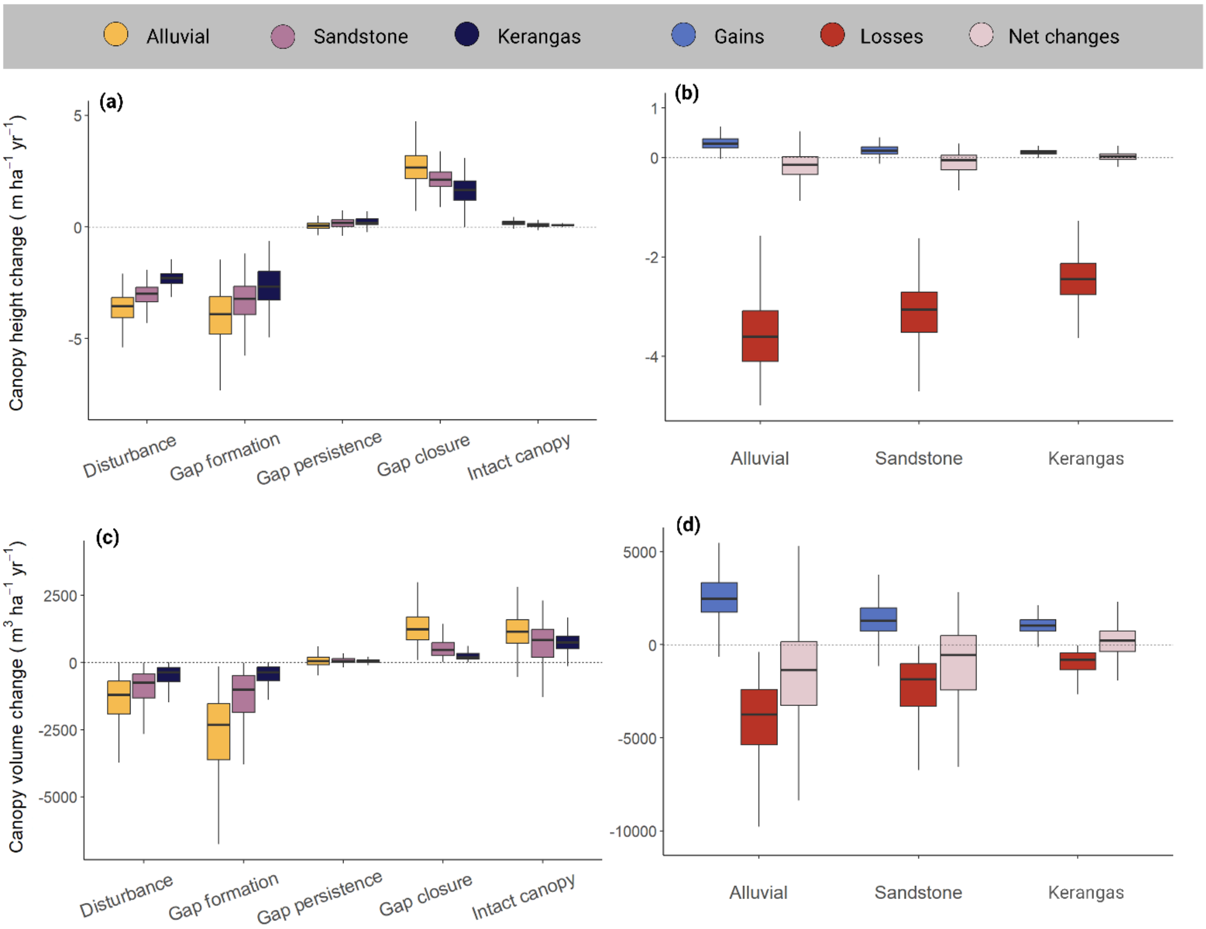

Fig. S7: Variation in canopy height and volume change across the three forest types using the alternative definition of canopy gaps and disturbances described in Methods S2. Boxplots on the top row show variation in height change, both (**a**) across the five canopy classes of canopy dynamic and (**b**) aggregated into canopy gains, losses and net changes. The bottom row shows the same results for canopy volume change. This figure is analogous to Fig. 4 in the main text.

Fig. S8 – A framework for classifying forest canopy dynamics incorporating lateral crown expansion

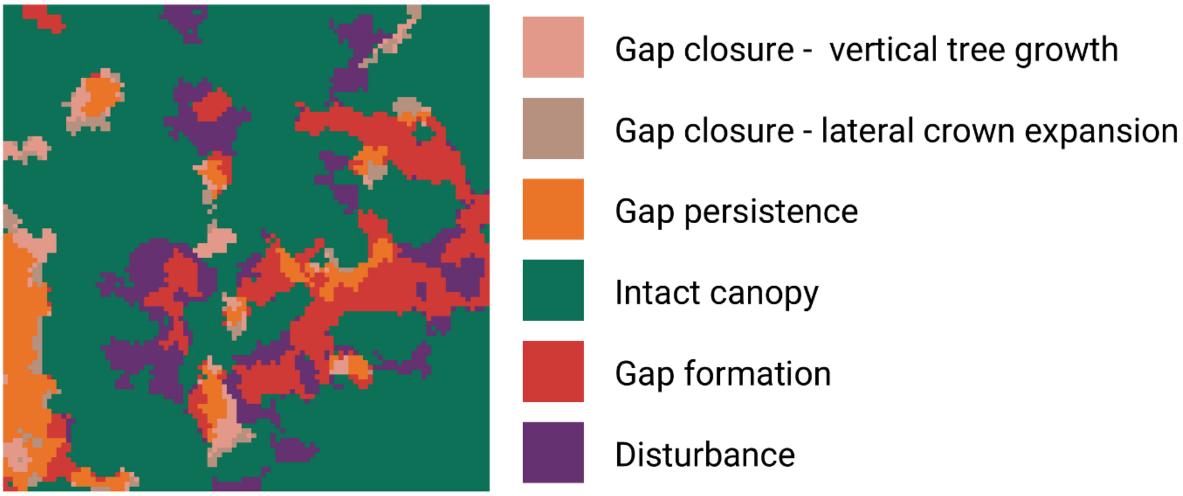

Fig. S8: Illustration of the framework used to classify forest canopy dynamics into various components of disturbance and growth, including canopy height gains driven by lateral crown expansion. For a description of the canopy dynamics classes see the main text and Fig. 2.

Fig. S9 – Variation in canopy dynamics rates incorporating lateral crown expansion

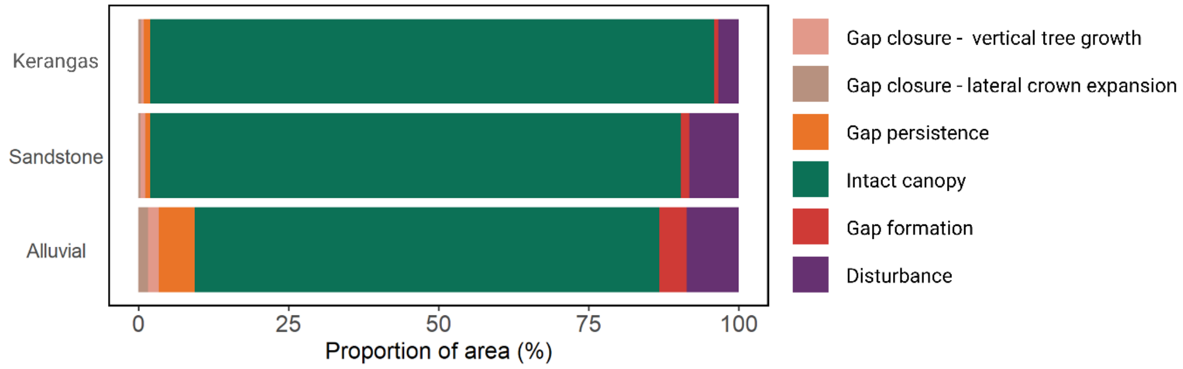

Fig. S9: Variation in rates of canopy dynamics across the three forest types, including those attributed to lateral crown expansion. Each bar corresponds to the proportion of the canopy area that either remained intact between 2014 and 2020 (green), was classified as a gap at both time steps (orange), underwent canopy closure resulting from vertical tree growth (pink) and lateral crown expansion (brown), or was subjected to a new disturbance (red and purple). For a description of the canopy dynamics classes see the main text and Fig. S8.

Fig. S10 – Variation in canopy height and volume change incorporating lateral crown expansion

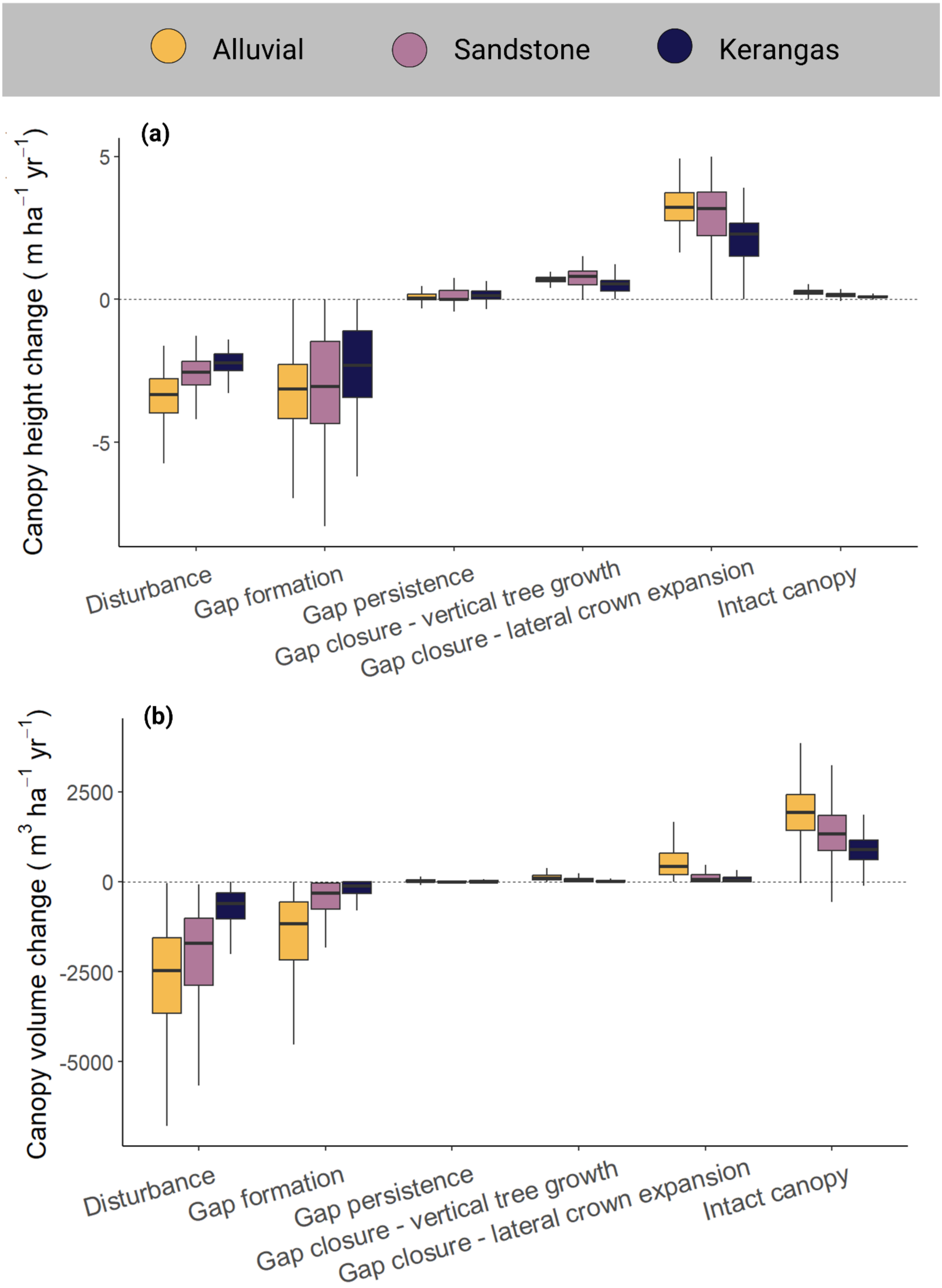

Fig. S10: Variation in canopy height and volume change across the three forest types, including those attributed to lateral crown expansion. For a description of the canopy dynamics classes see the main text and Fig. S8.

Methods S1 – Sensitivity of canopy dynamics to CHM algorithm and ground classification

To assess the sensitivity of canopy dynamics estimates to the approach used to generate 3D models of canopy surfaces, we compared results obtained using the locally adaptive spikefree CHMs described in the main text with two alternative approaches. First CHMs generated using a widely used Triangular Irregular Network (TIN) method (see Fischer et al. 2024 for details). Second a comparison based directly on digital surface models (DSMs) obtained using the spikefree algorithm, without normalising these in relation to ground heights from the DTMs. This second approach has the advantage that it removes potential errors in CHMs originating from differences in the ground classification accuracy among different ALS flights (although it is susceptible to noise due to systematic shifts in geopositioning that occur during ALS acquisition and post-processing). Both of these approaches gave results that were quantitatively very similar to those reported in the main text (Figs. S4-S5), suggesting that our results are robust to both the choice of algorithm used to generate the CHM and potential errors arising from the ground classification.

Methods S2 – Sensitivity of canopy dynamics to gap definition

To determine how sensitive estimates of canopy dynamics are to the way gaps are defined, we compared the approach presented in the main text (i.e., fixed height threshold of 10 m and minimum size threshold of 25 m^2) to one where we instead implemented a forest-type specific relative height threshold (areas <50% of mean canopy height within each forest type) and a size threshold of 10 m^2 to allow for smaller gaps in shorter forests. Using these criteria, we re-classified the canopy height models into the five dynamic categories described in the main text (i.e., gap formation, canopy disturbance, persistent gap, gap closure and intact canopy). In this case, canopy disturbances were defined as areas $\geqslant$10 m^2 where canopy height decreased by >25% of the forest-type-specific mean canopy height, but did not extend below 50% of this threshold and therefore did not meet our definition of a gap. This approach gave results that are quantitatively very similar to presented in the main text (Figs. S6-S7), indicating that our conclusions are not affected by the way we classify gaps in the ALS data.

Methods S3 – Spatial cross-validation of regression models

To robustly assess the predictive ability of the landscape-scale models relating variation in canopy gains and losses to topographic and canopy structural metrics, we used the spatial cross-validation developed by Ploton *et al.* (2020). This method explicitly accounts for spatial autocorrelation in the gridded data, which would otherwise substantially inflate estimates of model performance. Briefly, the method involves the following steps: (1) we used a semi-variogram to determine the scale at which the gridded data exhibited spatial autocorrelation, which we determined to be at around 1000 m; (2) we applied a buffered leave-one-out cross-validation approach where one grid cell is randomly selected for validation and all remaining grid cells that are >1000 m away from it are instead used to train the model; (3) we repeated this randomisation and model fitting step 1528 times to cover each 1-ha grid cell across the Sepilok landscape; and (4) we compared observed and predicted values of canopy volume gains and losses for the independent grid cells in each model run to calculate the proportion of explained variance (R^2) of the predictions.

Methods S4 – Drivers of gap closure: vertical growth and lateral crown expansion

Gap closure can result from both vertical tree growth associated with smaller tree cohorts in the understory and by lateral crown expansion of mature trees near gap edges. The latter is typically characterised by sudden large increases in heights in the CHMs that form a halo around the crowns of mature canopy trees, below which the canopy is often largely empty. To better understand the relative contribution of these two processes in driving the patterns of gap closure that we observe at Sepilok, we developed an approach to tease them apart. To do this we first identified core gap area in each forest type where we could confidently exclude the influence of lateral crown expansion. We then used the CHMs to calculate a realistic upper threshold to vertical height growth in each forest type, which we defined as the 90th percentile of height change between the two ALS surveys (1.4 m yr^{-1} in alluvial forests, 1.9 m yr^{-1} in sandstone forests and 1.3 m yr^{-1} in heath forests). Using this threshold, we were then able to attribute gap recovery to either vertical growth (pixel values below the height growth threshold) or crown expansion (pixel values greater than the potential height growth threshold; Figs. S8-S10).

Table S1 – Differences in canopy structure and dynamics among forest types

Table S1: Differences in topography, canopy structure and dynamics among the three forest types. Pair-wise differences among forest types were compared using ANOVAs and Tukey post-hoc tests.

**Topo-edaphic features**		**Alluvial vs sandstone**	**Alluvial vs kerangas**	**Sandstone vs kerangas**
Elevation (m)		-28 (*P*<0.001)	-61 (*P*<0.001)	-33 (*P*<0.001)
Soil phosphorus (mg kg^{-1})		+185 (*P*<0.001)	+210 (*P*<0.001)	+25 (*P*=0.003)
Topographic wetness index		+1.2 (*P*<0.001)	+1.2 (*P*<0.001)	+0 (*P*=1)
**Canopy 3D structures**		**Alluvial vs sandstone**	**Alluvial vs kerangas**	**Sandstone vs kerangas**
Mean canopy height (m)		-1.1 (*P*<0.001)	+6.1 (*P*<0.001)	+7.2 (*P*<0.001)
Maximum canopy height (m)		+5.1 (*P*<0.001)	+17.7 (*P*<0.001)	+12.6 (*P*<0.001)
Gap fraction at 10 m aboveground (%)		+7.4 (*P*<0.001)	+7.4 (*P*<0.001)	-0.03 (*P*=0.99)
Coefficient variation in canopy height		+0.13 (*P*<0.001)	+0.18 (*P*<0.001)	+0.05 (*P*<0.001)
**Canopy dynamics (5 classes)**		**Alluvial vs sandstone**	**Alluvial vs kerangas**	**Sandstone vs kerangas**
Disturbance	Canopy height change (m yr^{-1})	-0.8 (*P*<0.001)	-1.2 (*P*<0.001)	-0.4 (*P*<0.001)
	Volume change ($m^3ha^{-1}yr^{-1}$)	-940 (*P*<0.001)	-2286 (*P*<0.001)	-1346 (*P*<0.001)
	Area change (%)	+0.5 (*P*=0.2)	+5.3 (*P*<0.001)	+4.8 (*P*<0.001)
Gap formation	Canopy height change (m yr^{-1})	-0.4 (*P*<0.001)	-1.1 (*P*<0.001)	-0.7 (*P*<0.001)
	Volume change ($m^3ha^{-1}yr^{-1}$)	-1046 (*P*<0.001)	-1382 (*P*<0.001)	-336 (*P*<0.001)
	Area change (%)	+3.1 (*P*<0.001)	+3.9 (*P*<0.001)	+0.8 (*P*=0.001)
Gap persistence	Canopy height change (m yr^{-1})	-0.04 (*P*=0.007)	-0.07 (*P*<0.001)	-0.03 (*P*=0.2)
	Volume change ($m^3ha^{-1}yr^{-1}$)	+18 (*P*=0.01)	-4 (*P*=0.83)	-22 (*P*=0.02)
	Area change (%)	+5.2 (*P*<0.001)	+4.9 (*P*<0.001)	-0.4 (*P*=0.69)
Gap closure	Canopy height change (m yr^{-1})	+0.5 (*P*<0.001)	+0.8 (*P*<0.001)	+0.3 (*P*<0.001)
	Volume change ($m^3ha^{-1}yr^{-1}$)	+454 (*P*<0.001)	+533 (*P*<0.001)	+79 (*P*=0.04)
	Area change (%)	+2.2 (*P*<0.001)	+2.5 (*P*<0.001)	+0.3 (*P*=0.11)
Intact canopy	Canopy height change (m yr^{-1})	+0.1 (*P*<0.001)	+0.2 (*P*<0.001)	+0.1 (*P*<0.001)
	Volume change ($m^3ha^{-1}yr^{-1}$)	+614 (*P*<0.001)	+1069 (*P*<0.001)	+455 (*P*<0.001)
	Area change (%)	-11 (*P*<0.001)	-16.5 (*P*<0.001)	-5.6 (*P*<0.001)
**Canopy dynamics (3 classes)**		**Alluvial vs sandstone**	**Alluvial vs kerangas**	**Sandstone vs kerangas**
Gains	Canopy height change (m yr^{-1})	+0.1 (*P*<0.001)	+0.2 (*P*<0.001)	+0.1 (*P*<0.001)

	Volume change ($m^3 ha^{-1} yr^{-1}$)	+1086 (*P*<0.001)	+1597 (*P*<0.001)	+511 (*P*<0.001)
Losses	Canopy height change ($m\ yr^{-1}$)	+0.6 (*P*<0.001)	+1.1 (*P*<0.001)	+0.4 (*P*<0.001)
	Volume change ($m^3 ha^{-1} yr^{-1}$)	+1987 (*P*<0.001)	+3669 (*P*<0.001)	+1682 (*P*<0.001)
Net change	Canopy height change ($m\ yr^{-1}$)	-0.1 (*P*<0.001)	-0.2 (*P*<0.001)	-0.1 (*P*<0.001)
	Volume change ($m^3 ha^{-1} yr^{-1}$)	-901 (*P*<0.001)	-2072 (*P*<0.001)	-1171 (*P*<0.001)

Table S2 – Summary of regression models of canopy volume dynamics

Table S2: Summary of regression models assessing how canopy volume gains, losses, and net changes relate to elevation, TWI, H_{max} and H_{cv}. Variation across the three forest types was accounted for by including an interaction term with forest type in each model. For each model, fit statistics (R^2) and *P*-values associated with each term are reported. R^2 values were calculated using the spatial cross-validation approach described in Methods S3.

**Models**	**Predictors**	***P*-values**	**R^2**
Gains ~ H_{max}	H_{max}	$P < 0.001$	0.24
Gains ~ H_{max} × Soil type	H_{max}	$P < 0.001$	0.33
	Soil type	$P < 0.001$	
	H_{max} × Soil type	$P < 0.001$	
Gains ~ H_{cv}	H_{cv}	$P < 0.001$	0.50
Gains ~ H_{cv}× Soil type	H_{cv}	$P < 0.001$	0.51
	Soil type	$P < 0.001$	
	H_{cv} × Soil type	$P < 0.001$	
Gains ~ Elevation	Elevation	$P < 0.001$	0.16
Gains ~ Elevation × Soil type	Elevation	$P < 0.001$	0.27
	Soil type	$P < 0.001$	
	Elevation × Soil type	$P < 0.001$	
Gains ~ TWI	TWI	$P < 0.001$	0.08
Gains ~ TWI × Soil type	TWI	$P < 0.001$	0.28
	Soil type	$P < 0.001$	
	TWI × Soil type	$P < 0.001$	
Losses ~ H_{max}	H_{max}	$P < 0.001$	0.42
Losses ~ H_{max}× Soil type	H_{max}	$P < 0.001$	0.46
	Soil type	$P < 0.001$	
	H_{max} × Soil type	$P < 0.001$	
Losses ~ H_{cv}	H_{cv}	$P < 0.001$	0.21
Losses ~ H_{cv} × Soil type	H_{cv}	$P < 0.001$	0.38
	Soil type	$P < 0.001$	
	H_{cv} × Soil type	$P < 0.001$	
Losses ~ Elevation	Elevation	$P < 0.001$	0.34
Losses ~ Elevation × Soil type	Elevation	$P < 0.001$	0.36
	Soil type	$P < 0.001$	
	Elevation × Soil type	$P = 0.006$	
Losses ~ TWI	TWI	$P < 0.001$	0.04
Losses ~ TWI × Soil type	TWI	$P < 0.001$	0.32
	Soil type	$P < 0.001$	
	TWI × Soil type	$P = 0.04$	
Net changes ~ H_{max}	H_{max}	$P < 0.001$	0.09
Net changes ~ H_{max} × Soil type	H_{max}	$P < 0.001$	0.08
	Soil type	$P = 0.02$	
	H_{max} × Soil type	$P = 0.04$	
Net changes ~ H_{cv}	H_{cv}	$P < 0.001$	0.003
Net changes ~ H_{cv} × Soil type	H_{cv}	$P < 0.001$	0.04
	Soil type	$P < 0.001$	

	H_{cv} × Soil type	$P = 0.75$	
Net changes ~ Elevation	Elevation	$P < 0.001$	0.06
Net changes ~ Elevation × Soil type	Elevation	$P < 0.001$	0.04
	Soil type	$P = 0.002$	
	Elevation × Soil type	$P = 0.36$	
Net changes ~ TWI	TWI	$P < 0.001$	0.004
Net changes ~ TWI × Soil type	TWI	$P < 0.001$	0.04
	Soil type	$P < 0.001$	
	TWI × Soil type	$P = 0.51$	
Gains ~ Elevation × Soil type + TWI × Soil type	Elevation	$P < 0.001$	0.28
	TWI	$P < 0.001$	
	Soil type	$P < 0.001$	
	Elevation × Soil type	$P < 0.001$	
	TWI × Soil type	$P = 0.003$	
Losses ~ Elevation × Soil type + TWI × Soil type	Elevation	$P < 0.001$	0.37
	TWI	$P = 0.10$	
	Soil type	$P < 0.001$	
	Elevation × Soil type	$P = 0.01$	
	TWI × Soil type	$P = 0.05$	
Gains ~ H_{max} × Soil type + H_{cv} × Soil type + Elevation × Soil type + TWI × Soil type	H_{max}	$P < 0.001$	0.53
	H_{cv}	$P < 0.001$	
	Elevation	$P < 0.001$	
	TWI	$P = 0.02$	
	Soil type	$P < 0.001$	
	H_{max} × Soil type	$P < 0.001$	
	H_{cv} × Soil type	$P < 0.001$	
	Elevation × Soil type	$P = 0.23$	
	TWI × Soil type	$P = 0.05$	
Losses ~ H_{max} × Soil type + H_{cv} × Soil type + Elevation × Soil type + TWI × Soil type	H_{max}	$P < 0.001$	0.44
	H_{cv}	$P < 0.001$	
	Elevation	$P < 0.001$	
	TWI	$P = 0.69$	
	Soil type	$P < 0.001$	
	H_{max} × Soil type	$P = 0.04$	
	H_{cv} × Soil type	$P = 0.05$	
	Elevation × Soil type	$P = 0.04$	
	TWI × Soil type	$P = 0.39$	

Reference

Fischer, F.J., Jackson, T.D., Vincent, G. & Jucker, T. 2024. Robust characterisation of forest structure from airborne laser scanning—A systematic assessment and sample workflow for ecologists. *Methods in Ecology and Evolution,* 15**,** 1873-1888.

Ploton, P., Mortier, F.d.r., Réjou-Méchain, M., Barbier, N., Picard, N., Rossi, V., Dormann, C., Cornu, G., Viennois, G.l., Bayol, N., et al. 2020. Spatial validation reveals poor predictive performance of large-scale ecological mapping models. *Nature Communications,* 11**,** 4540.
